# Circulatory health risks from additive multi-pollutant models: short-term exposure to three common air pollutants in Canada

**DOI:** 10.1007/s11356-022-22947-4

**Published:** 2022-09-29

**Authors:** Hwashin Hyun Shin, James Owen, Aubrey Maquiling, Rajendra Prasad Parajuli, Marc Smith-Doiron

**Affiliations:** 1 Environmental Health Science and Research Bureau, Health Canada, 269 Laurier Ave. W., ON Ottawa, Canada; 2 Department of Mathematics and Statistics, Queen’s University, ON Kingston, Canada; 3 Central Department of Zoology, Central Campus, Institute of Science & Technology (IOST), Tribhuvan University, Kritipur-1, Kathmandu, Nepal

**Keywords:** Fine particulate matter (PM_2.5_), Ground-level ozone, Hospitalization, Mortality, Multi-pollutant, Nitrogen dioxide (NO_2_)

## Abstract

Numerous studies have reported adverse health effects of ambient air pollution on circulatory health outcomes mainly based on single-pollutant models. However, limited studies have focused on adjusted effect of multi-pollutant exposures on public health. This study aimed to examine short-term effects of three common air pollutants—ground-level ozone (ozone), nitrogen dioxide (NO_2_), and fine particulate matter (PM_2.5_)—through multi-pollutant models for mixed effect of adjustment. Daily data (circulatory hospitalization and mortality) and hourly data (air pollutants and temperature) were collected for 24 Canadian cities for 2001–2012. We applied generalized additive over-dispersion Poisson regression models with 1, 2, or 3 pollutants for city-specific risks, and Bayesian hierarchical models for national risks. This study found little mixed effect of adjustment through multi-pollutant models (ozone and/or NO_2_ and/or PM_2.5_) for circulatory hospitalization or mortality in Canada for 2001–2012, indicating that the 1-pollutant model did not result in considerable under- or over-estimates. It seemed weak-to-moderate correlations among air pollutants did not change the significant effect of one air pollutant after accounting for others. Inconsistent findings between other previous studies and this study indicate the need of comparable study design for multi-pollutant effect analysis.

**Supplementary Information:**

The online version contains supplementary material available at 10.1007/s11356-022-22947-4.

## Introduction

Numerous epidemiologic studies have reported adverse health effects of ambient air pollution on morbidity and mortality. Among various air pollutants and health outcomes, it was commonly observed across countries that air pollutants such as ground-level ozone, nitrogen dioxide (NO_2_), and fine particulate matter of diameter less than 2.5 µm (PM_2.5_) were associated with circulatory-related health outcomes such as hospitalization and mortality (Shin et al. 2021, 2020, 2012; Rodríguez-Villamizar et al. 2018; Zhang et al. 2017a; Dong et al. 2013; Linares et al. 2010; Zhang et al. 2006; Poloniecki et al 1997). These three air pollutants are included in the WHO Global Air Quality Guidelines, which offers global guidance on thresholds and limits for key air pollutants associated with various public health risks (WHO 2021). Many countries also set up national guidance and standards for the three air pollutants, for example: the Canadian Ambient Air Quality Standards, the National Ambient Air Quality Standards (NAAQS) in the USA and Mexico, the European Commission’s Technical Air Quality Standards, and the NAAQS in China, India, and Japan in Asia. For the mutual adjustment among the three air pollutants, multi-pollutant models have been employed to estimate their associations with circulatory health risk for short- or long-term exposure. However, previous studies have reported inconsistent mixed effect of adjustment (i.e., effect was not in the same line) for circulatory health risks through 2- or 3-pollutant models.

For circulatory hospitalization, a recent study in China during 2016–2018 (Jiang et al. 2020) reported that the effect of ozone on circulatory outpatient visits was increased after adjusting for PM_2.5_, when the concentration of ozone was higher than 100 μg/L. In a study of Colombian cities, the effect of PM_2.5_ on circulatory hospitalization was increased after adjusting for NO_2_ in a two-pollutant model during 2011–2014 (Rodríguez-Villamizar et al. 2019). Some studies have reported examples of the three pollutants having stable risks after adjustment for a second pollutant for hospitalizations due to circulatory system disease (Gu et al. 2020) and stroke (Cruz et al. 2015), while others report unstable or insignificant associations, after adjustment for other pollutants, for cardiac disease (Barnett et al. 2006), cardiovascular disease (CVD) (Franck et al. 2014), and circulatory disease (Guo et al. 2018). A mix of stable and decreased associations, depending on the pollutant, has been observed for CVD (Moolgavkar 2000), stroke (Tsai et al. 2003; Tian et al. 2018), and cardio-cerebrovascular disease (Wang et al. 2019).

In contrast, more comparable results have been reported from multi-pollutant models for circulatory mortality. In 2-pollutant models, adjustment for another pollutant did not mitigate the short-term risks of the three pollutants’ associations with circulatory mortality (Cheng et al. 2016; Costa et al. 2017) and CVD mortality (Mazidi and Speakman 2018; Zhang et al. 2019). In another study, the risk of CVD mortality associated with ozone was reported stable with PM_10_ and SO_2_, but not NO_2_ (Zhang et al. 2017a)

In 3-pollutant models, risk of circulatory mortality associated with ozone has been reported robust to mutual adjustment for PM_2.5_ and NO_2_ in the USA (Turner et al. 2016), and ozone and PM_10_ in Canada (Lippmann et al. 2000). In China, associations with PM_2.5_ remained after adjustment for NO_2_ and ozone (Qu et al. 2018), and ozone and SO_2_ (including adjustment for collinearity; Mokoena et al. 2019). Similarly, in Madrid, relative risks of acute myocardial infarction mortality attributable to PM_2.5_ remained significant after adjusting for ozone and NO_2_ (Maté et al. 2010). Two studies reported decreased associations between NO_2_ and CVD mortality after adjustment for SO_2_ and PM_10_ (Zhang et al. 2017b), and CO and PM_10_ (Yang et al. 2017).

Taken together, existing evidence from multi-pollutant models on the association between air pollution and circulatory hospitalization and mortality is limited and inconclusive. The inconsistency among the previous studies could be related to various factors: for example, statistical models (e.g., different assumptions and confounders), health care systems (e.g., availability and medical insurance system), study population (e.g., age group and residence location), study period, localized degree of correlation between pollutants, and environmental backgrounds (e.g., weather and green space). In this study, we aimed to identify under- or over-estimates from single-pollutant models compared to multi-pollutant models with 2 or 3 pollutants for circulatory disease in Canada. We estimated the adverse health effects of short-term exposures to the three major air pollutants (ozone, NO_2_, and PM_2.5_) on circulatory health outcomes (mortality and hospitalizations) in additive modeling. We further investigated if the overall associations were influenced by season, considering warm (April to September) and cold (October to March) season associations in addition to year-round. We also examined the correlations among the three air pollutants and investigated if this was a key factor to meaningful changes (significant to insignificant association, or vice versa) from single-versus multi-pollutant models.

## Materials and methods

### Study design

The study is designed to estimate adverse health effects of short-term exposure to three air pollutants (ozone, NO_2_, and PM_2.5_) on two circulatory health outcomes (hospitalization and mortality) through 1-, 2-, and 3-pollutant models. Ozone and NO_2_ data are available for 29 years (1984–2012), and PM_2.5_ data for 12 years (2001–2012). Mortality data is also available for 29 years (1984–2012), whereas hospitalization data is available for 17 years (1996–2012). Taken all together, the study period is set for 2001 to 2012.

For spatial coverage of the study, 24 urban census divisions (CDs) are selected mainly for three reasons: large population size, availability of multiple ground-monitoring stations for reliable measurements of air pollution concentrations, and their associated CDs that are considered stable during the study period (Figure S1 in Online Resource). The 24 CDs (study population) represented about 52% of the total Canadian population (target population) located in 8 out of 10 provinces in Canada: Newfoundland and Labrador (St. John’s), Nova Scotia (Halifax), Quebec (Montreal), Ontario (Ottawa, Durham, York, Toronto, Peel, Halton, Hamilton, Niagara, Waterloo, Windsor, Sarnia, London, Sudbury, and Sault Ste. Marie), Manitoba (Winnipeg), Saskatchewan (Regina and Saskatoon), Alberta (Calgary and Edmonton), and British Columbia (Vancouver).

For short-term exposure, this study defines it as a 2-week time window prior to death or hospitalization. This definition is arbitrary but necessary to separate short-term exposure effects from long-term exposure effects such as seasonal effect, even though there has been no consensus on the short-term period. This process can be done by a smoother on calendar time (see the “Statistical models” section for more details).

For the multi-pollutant models, this study examines three 2-pollutant models (ozone/NO_2_, ozone/PM_2.5_, and NO_2_/PM_2.5_) and a 3-pollutant model (ozone/NO_2_/PM_2.5_), compared to three single-pollutant models (base model). Considering seasonal changes in correlations among the pollutants and their relationships with circulatory health outcomes, this study examines all models by season: warm (April to September), cold (October to March), and year-round (base).

### Data sources and analysis procedures

This study is based on four databases on daily air pollution, temperature, hospitalization, and mortality. First, all hourly air pollution data were obtained from the National Air Pollution Surveillance System (NAPS), maintained by Environment and Climate Change Canada (ECCC) (ECCC 2013). A total of 120 NAPS stations were used to cover the 24 CDs. The hourly data were converted to 8-h maximum for ozone, and to 24-h average for NO_2_ and PM_2.5_ for each monitoring station, following the metrics used for the Canadian Ambient Air Quality Standards, which were developed by the Canadian Council of Ministers of the Environment. Multiple stations within the same CD were averaged to represent daily concentrations for the CD. Second, daily temperature data was obtained from the National Climate Data and Information Archive of ECCC, as it was an important confounder in the association between air pollutants and circulatory health outcomes. A total of 250 weather stations were used, and multiple weather stations in the same CD were averaged to represent daily temperature for the CD. Third, circulatory hospitalization data was obtained from the Canadian Institute for Health Information (CIHI 2018). Fourth, daily counts of circulatory mortality were obtained from the Canadian Vital Statistics Death Database managed by Statistics Canada (Statistics Canada 2019). Circulatory health outcomes include diseases defined in section I00-I99 of the International Classification of Diseases 10^th^ Revision (ICD-10, WHO 2019). For hospitalizations in some CDs, both versions ICD-9 and ICD-10 were used based on a conversion table provided by the CIHI. Daily counts of circulatory hospitalization and mortality were aggregated at the CD level.

### Statistical models

A two-stage model was employed to first estimate the CD-specific associations between air pollutants and health outcomes, and then pool the CD-specific associations to represent the nationwide association. In the first stage, the daily hospitalization or mortality counts were modeled against daily ozone, NO_2_, and PM_2.5_ concentrations. A generalized additive Poisson regression model with multi-pollutants was applied to individual CDs for location *i*, season *j*, pollutant *k*, day $t$ during the study period, and lag day $l$ as follows (Eq. [1]):

1 $$\begin{array}{c}\mathrm{log}\left(E\left[{Y}_{ij}\left(t\right)\right]\right)={\beta }_{0}+{\sum }_{k=1}^{n}{\beta }_{1\mathrm{ijk}}\left(t\right)*{x}_{ijk}\left(t-l\right)+{f}_{ij}\left(t\right)+{g}_{ij}\left(temp\left(t\right)\right)+{DOW}_{ij}\left(t\right),\end{array}$$

where ${Y}_{ij}(t)$, ${x}_{ijk}(t)$ and ${DOW}_{ij}\left(t\right)$ are daily hospitalization or mortality count (the response variables), daily air pollutant concentrations (the predictor), and the day of week (a confounder), respectively, in linear relationship. Two more confounders, ${f}_{ij}\left(t\right)$ and ${g}_{ij}\left(t\right),$ are non-linear smoothing functions for calendar time (*t* = *1,2,3,…, T*) and temperature ($temp\left(t\right)$), respectively. We used a smoother (natural cubic splines) on the temperature with degree of freedom 3 to capture a U-shape relationship between temperature and health outcomes, which indicates stronger effect on mortality from cold and hot temperatures. In particular, the parameter ${\beta }_{1ijk}$ for $k$-pollutant models (*k* = 1,2,3) for 0- to 6-day lagged exposures with the same lag structure for each pollutant are of interest to be estimated, indicating the effects of ozone, NO_2_, and/or PM_2.5_ on the circulatory hospitalization or mortality in log scale.

In the second stage, a hierarchical Bayesian approach was used to obtain national risk estimates from the CD-specific risk associations for all years together, 2001–2012. More detail on this approach is available in previous papers (Shin et al. 2009; Shin et al. 2012). The national estimates were reported for 0- to 6-day lagged effects as ${1000*\beta }_{1\mathrm{i}jk}(t-l)$ with 95% posterior intervals, which indicates the relative risk as percent change in health outcomes per 10 unit change in each air pollutant following the Taylor series approximation for small ${\beta }_{1\mathrm{i}jk}$. The statistical computing language and environment R 4.0.3 was used for all computations (R Core Team 2020).

## Results

### Circulatory hospitalization and mortality

Table 1 presents annual average rates of circulatory hospitalization and mortality as ratios of both the study population and all non-accidental hospitalizations and mortalities. Circulatory hospitalizations occurred at a rate of approximately 1.0% (range, 0.4–1.6%) of the study population and accounted for 15.1% (range, 11.6–19.2%) of non-accidental hospitalizations, with the highest ratios occurring in relatively small CDs— Sault Ste. Marie and Sarnia, respectively. Circulatory mortality occurred at rates of approximately 0.2% (range, 0.1–0.4%) of the study population and roughly 32.9% (range, 28.1–39.3%) of non-accidental mortalities, with the highest ratios being in Sarnia. The overall rate of circulatory hospitalizations was larger than the mortality rate by a factor of five when compared to the study population (1.0 vs. 0.2%). In contrast, the overall mortality rate was roughly double the hospitalization rate when compared to non-accidental health outcomes (33% vs. 15%). This indicates circulatory mortality took a relatively large portion of the all-cause mortality.

**Table 1:** Annual average of circulatory hospitalization and mortality rates from 2001 to 2012

City^a^	Hospitalization in % (SD)		Mortality in % (SD)	
	% ratio^b^	% to all cause^c^	% ratio^d^	% to all cause^e^
Halifax	0.8 (0.06)	14.4 (0.90)	0.2 (0.07)	31.2 (0.97)
Saint John	1.4 (0.14)	16.7 (1.15)	0.4 (0.16)	33.6 (1.14)
Quebec City	1.5 (0.06)	13.5 (0.69)	0.2 (0.16)	28.1 (1.73)
Montreal	1.2 (0.05)	12.3 (0.51)	0.2 (0.10)	29.7 (1.17)
Ottawa	0.7 (0.02)	14.6 (0.28)	0.2 (0.06)	32.2 (0.63)
Durham	0.6 (0.05)	15.0 (0.55)	0.2 (0.10)	31.1 (0.75)
York	0.4 (0.02)	14.8 (0.24)	0.1 (0.03)	30.2 (1.01)
Toronto	0.9 (0.03)	16.0 (0.21)	0.2 (0.07)	31.3 (0.37)
Peel	0.5 (0.04)	14.3 (0.37)	0.1 (0.05)	30.3 (0.60)
Halton	0.7 (0.03)	15.0 (0.52)	0.2 (0.12)	30.1 (1.12)
Hamilton	1.2 (0.03)	18.5 (0.55)	0.2 (0.14)	32.2 (1.54)
Niagara	1.0 (0.08)	16.9 (0.36)	0.3 (0.16)	36.0 (0.72)
Waterloo	0.7 (0.07)	13.7 (0.44)	0.2 (0.08)	33.4 (0.51)
Windsor	1.1 (0.04)	15.7 (0.64)	0.3 (0.13)	37.0 (0.53)
Sarnia	1.4 (0.08)	19.2 (0.82)	0.4 (0.23)	39.3 (1.87)
London	0.8 (0.06)	13.5 (0.98)	0.2 (0.06)	30.3 (0.63)
Sudbury	1.3 (0.08)	18.1 (0.50)	0.3 (0.14)	33.8 (0.87)
Sault Ste. Marie	1.6 (0.08)	17.9 (0.73)	0.3 (0.11)	32.7 (0.92)
Winnipeg	0.9 (0.05)	15.2 (0.45)	0.3 (0.10)	34.0 (0.85)
Regina	1.2 (0.07)	13.7 (0.70)	0.2 (0.10)	32.9 (0.94)
Saskatoon	1.0 (0.08)	14.7 (0.68)	0.2 (0.11)	34.1 (0.77)
Calgary	0.7 (0.04)	11.9 (0.45)	0.2 (0.08)	37.1 (1.01)
Edmonton	0.7 (0.04)	11.6 (0.42)	0.2 (0.06)	34.5 (0.92)
Vancouver	0.8 (0.02)	14.0 (0.20)	0.2 (0.06)	33.8 (1.13)
**Combined**^**f**^	**1.0 (0.00)**	**15.1 (0.02)**	**0.2 (0.00)**	**32.9 (0.03)**

^a^Cities are ordered geographically from east to west

^b^Study hospitalization counts/Study population) × 100

^c^Circulatory hospitalization counts/ non-accidental hospitalization counts) × 100

^d^Study mortality counts/Study population) × 100

^e^Circulatory mortality counts/non-accidental mortality counts) × 100

^f^Non-weighted average over 24 cities

### Concentrations of three air pollutants

Online Resource Table S1 presents seasonal (warm, cold) average temperature and air pollutant (ozone, NO_2_, PM_2.5_) concentrations for 2001–2012. Average temperatures were 15 °C (range, 12–18 °C) during the warm season and -1 °C (range, − 8–6 °C) during the cold season. Overall, the ozone concentrations were higher in the warm season than the cold season (38 ppb (range, 26–47 ppb) vs. 27 ppb (range, 21–32 ppb)), with somewhat higher city-to-city variation during the warm season. On average, Windsor had the highest warm season ozone concentrations (47 ppb), while the highest cold season ozone concentration (32 ppb) was shared among four cities (St. John’s, York, Sudbury, Sault Saint Marie). Similarly, PM_2.5_ concentrations were also higher in the warm season than the cold season (8 vs. 6 µg/m^3^). Sarnia recorded the highest concentrations of PM_2.5_ in both warm and cold seasons (13 and 10 µg/m^3^, respectively). In contrast, NO_2_ was lower in the warm season than the cold season (10 vs. 15 ppb). The highest NO_2_ concentrations were observed in Toronto during the warm season (18 ppb) and Calgary during the cold season (25 ppb). Taken together, the highest average air pollutant concentrations depend on season and location.

### Correlations among the three air pollutants

During the study period, the correlations among the three air pollutants varied across locations and seasons but the annual correlations were relatively stable over time as summarized in Table S2 and Figure S2. Higher correlations (0.7 ~ 0.8) were found between ozone and PM_2.5_ during the warm season mainly in the province of Ontario, and between PM_2.5_ and NO_2_ during the cold season mainly in the province of Quebec. In contrast, the correlation between ozone and NO_2_ was low overall: 0.2 (CD-to-CD range, − 0.1 to 0.5) in the warm season and − 0.2 (− 0.5 to 0) in the cold season. The correlations between ozone and PM_2.5_ showed more prominent changes by season: as high as 0.7 for 9 CDs in the warm season with an overall correlation of 0.5 (0.2 to 0.7); but as low as − 0.1 (− 0.6 to 0.1) in the cold season. However, the correlations between NO_2_ and PM_2.5_ were always positive, and higher in the cold season, 0.5 (0.1 to 0.8), than the warm season, 0.3 (0.0 to 0.7). As expected, the year-round correlations appeared smaller than seasonal correlations.

### Multi-pollutant associations between ozone and circulatory hospitalization and mortality

**Fig. 1:**
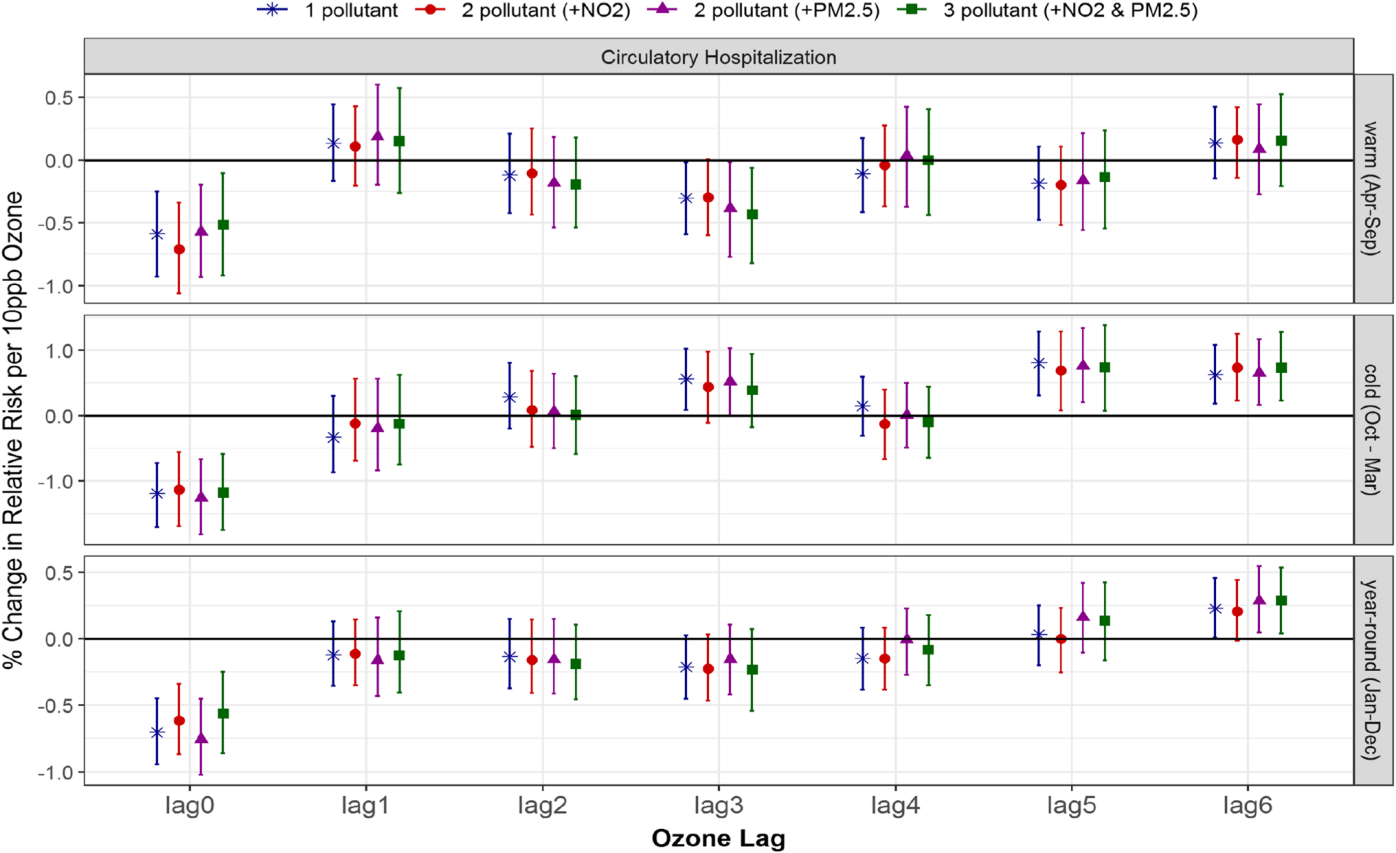
Comparison of estimated associations with 95% credible intervals between ozone and circulatory hospitalization from multi-pollutant models by season and lag: (*) 1-pollutant model, (●) 2-pollutant model, (▲) another 2-pollutant model, and (■) 3-pollutant model; 3 seasons of warm (Apr to Sept), cold (Oct to Mar) and year-round (Jan to Dec); and (c) 7 lags of 0- to 6-day lagged air pollutant

Associations between ozone and circulatory hospitalization generally increased with the air pollutants’ lag. While there were no significant associations between ozone and circulatory hospitalization during the warm season, we found significant associations for both 5- and 6-day lagged ozone during the cold season in both single- and multi-pollutant models adjusted for NO_2_ and PM_2.5_ (Fig. 1 and Table 2). The magnitudes of the associations are comparable for all models, with 5-day lagged ozone having risk estimates of 0.8% with 95% posterior interval (0.3–1.3) for the 1-pollutant model compared to the 2-pollutant model, 0.7% (0.1, 1.3) with NO_2_ and 0.8% (0.2, 1.3) with PM_2.5_, and to the 3-pollutant model with ozone, NO_2_, and PM_2.5,_ 0.7% (0.1, 1.4). In contrast, the association between 3-day lagged ozone and circulatory hospitalization was significant in the single-pollutant model, 0.6% (0.1, 1.0); however, it became insignificant with the addition of NO_2_ or PM_2.5_ in the multi-pollutant models.

**Table 2:** % change in risk of circulatory hospitalization per 10 unit increase in air pollutant

	Season^a^	Model	Air pollutant day lag						
			Lag 0	Lag 1	Lag 2	Lag 3	Lag 4	Lag 5	Lag 6
Ozone	Warm	1 pollutant	− 0.6% (− 0.9, − 0.2)	0.1% (− 0.2, 0.4)	− 0.1% (− 0.4, 0.2)	− 0.3% (− 0.6, 0.0)	− 0.1% (− 0.4, 0.2)	− 0.2% (− 0.5, 0.1)	0.1% (− 0.1, 0.4)
		2 pollutant (+ NO_2_)	− 0.7% (− 1.1, − 0.3)	0.1% (− 0.2, 0.4)	− 0.1% (− 0.4, 0.3)	− 0.3% (− 0.6, 0.0)	0.0% (− 0.4, 0.3)	− 0.2% (− 0.5, 0.1)	0.2% (− 0.1, 0.4)
		2 pollutant (+ PM2.5)	− 0.6% (− 0.9, − 0.2)	0.2% (− 0.2, 0.6)	− 0.2% (− 0.5, 0.2)	− 0.4% (− 0.8, 0.0)	0.0% (− 0.4, 0.4)	− 0.2% (− 0.6, 0.2)	0.1% (− 0.3, 0.4)
		3 pollutant (+ NO_2_ & PM2.5)	− 0.5% (− 0.9, − 0.1)	0.1% (− 0.3, 0.6)	− 0.2% (− 0.5, 0.2)	− 0.4% (− 0.8, − 0.1)	0.0% (− 0.4, 0.4)	− 0.1% (− 0.5, 0.2)	0.2% (− 0.2, 0.5)
	cold	1 pollutant	− 1.2% (− 1.7, (− 0.7)	− 0.3% (− 0.9, 0.3)	0.3% (− 0.2, 0.8)	**0.6% (0.1, 1.0 **^**b**^	0.1% (− 0.3, 0.6)	*0.8% (0.3, 1.3)*	*0.6% (0.2, 1.1)*
		2 pollutant (+ NO2)	− 1.1% (− 1.7, (− 0.6)	− 0.1% (− 0.7, 0.6)	0.1% (− 0.5, 0.7)	0.4% (− 0.1, 1.0)	− 0.1% (− 0.7, 0.4)	*0.7% (0.1, 1.3)*	*0.7% (0.2, 1.3)*
		2 pollutant (+ PM2.5)	− 1.3% (− 1.8, − 0.7)	− 0.2% (− 0.8, 0.6)	0.1% (− 0.5, 0.6)	0.5% (− 0.0, 1.0)	0.0% (− 0.5, 0.5)	*0.8% (0.2, 1.3)*	*0.7% (0.2, 1.2)*
		3 pollutant (+ NO2 & PM2.5)	− 1.2% (− 1.7, − 0.6)	− 0.1% (− 0.8, 0.6)	0.0% (− 0.6, 0.6)	0.4% (− 0.2, 0.9)	− 0.1% (− 0.6, 0.4)	*0.7% (0.1, 1.4)*	*0.7% (0.2, 1.3)*
	year-round	1 pollutant	− 0.7% (− 0.9, − 0.4)	− 0.1% (− 0.4, 0.1)	− 0.1% (− 0.4, 0.2)	− 0.2% (− 0.5, 0.0)	− 0.1% (− 0.4, 0.1)	0.0% (− 0.2, 0.2)	*0.2% (0.0, 0.5)*
		2 pollutant (+ NO_2_)	− 0.6% (− 0.9, − 0.3)	− 0.1% (− 0.4, 0.1)	− 0.2% (− 0.4, 0.1)	− 0.2% (− 0.5, 0.0)	− 0.1% (− 0.4, 0.1)	0.0% (− 0.3, 0.2)	*0.2% (− 0.0, 0.4)*
		2 pollutant (+ PM2.5)	− 0.8% (− 1.0, − 0.4)	− 0.2% (− 0.4, 0.2)	− 0.2% (− 0.4, 0.1)	− 0.2% (− 0.4, 0.1)	0.0% (− 0.3, 0.2)	0.2% (− 0.1, 0.4)	*0.3% (0.0, 0.5)*
		3 pollutant (+ NO_2_ & PM2.5)	− 0.6% (− 0.9, − 0.2)	− 0.1% (− 0.4, 0.2)	− 0.2% (− 0.5, 0.1)	− 0.2% (− 0.5, 0.1)	− 0.1% (− 0.4, 0.2)	0.1% (− 0.2, 0.4)	*0.3% (0.0, 0.5)*
NO2	warm	1 pollutant	*1.8% (0.7, 3.0)*^*c*^	− 0.7% (− 1.8, 0.3)	− 0.3% (− 1.0, 0.4)	− 0.5% (− 1.3, 0.4)	− 0.8% (− 1.4, − 0.1)	0.0% (− 0.8, 0.7)	0.2% (− 0.8, 1.1)
		2 pollutant (+ ozone)	*2.1% (0.9, 3.3)*	− 0.8% (− 1.9, 0.2)	− 0.3% (− 1.0, 0.5)	− 0.3% (− 1.2, 0.6)	− 0.8% (− 1.5, − 0.1)	0.0% (− 0.7, 0.8)	0.1% (− 0.9, 1.1)
		2 pollutant (+ PM2.5)	*2.5% (1.2, 3.9)*	− 0.8% (− 1.9, 0.3)	− 0.2% (− 1.0, 0.6)	− 0.4% (− 1.4, 0.6)	− 0.5% (− 1.4, 0.2)	0.1% (− 0.7, 0.9)	0.1% (− 1.1, 1.1)
		3 pollutant (+ ozone & PM2.5)	*2.5% (1.2, 3.7)*	− 0.7% (− 2.0, 0.7)	− 0.3% (− 1.0, 0.4)	− 0.4% (− 1.3, 0.6)	− 0.5% (− 1.3, 0.2)	0.2% (− 0.6, 1.1)	0.1% (− 1.0, 1.1)
	cold	1 pollutant	**0.8% (0.1, 1.4)**	**0.6% (0.1, 1.2)**	− 0.3% (− 0.9, 0.2)	− 0.6% (− 1.1, − 0.1)	− 0.6% (− 1.1, − 0.1)	− 0.7% (− 1.2, − 0.2)	− 0.1% (− 0.6, 0.4)
		2 pollutant (+ ozone)	0.2% (− 0.5, 0.8)	0.6% (− 0.0, 1.1)	− 0.3% (− 1.0, 0.3)	− 0.3% (− 0.9, 0.2)	− 0.6% (− 1.2, 0.0)	− 0.3% (− 0.9, 0.3)	0.3% (− 0.3, 0.8)
		2 pollutant (+ PM2.5)	**0.9% (0.1, 1.7)**	0.6% (− 0.1, 1.2)	− 0.2% (− 0.9, 0.4)	− 0.6% (− 1.2, 0.1)	− 0.4% (− 1.1, 0.3)	− 0.6% (− 1.3, 0.1)	0.0% (− 0.6, 0.7)
		3 pollutant (+ ozone & PM2.5)	0.8% (− 0.5, 1.9)	0.3% (− 0.6, 1.3)	− 0.1% (− 1.1, 0.8)	− 0.4% (− 1.4, 0.5)	− 0.6% (− 1.5, 0.4)	− 0.4% (− 1.3, 0.6)	0.4% (− 0.6, 1.3)
	year-round	1 pollutant	*1.1% (0.5, 1.6)*	0.4% (− 0.1, 0.8)	− 0.2% (− 0.6, 0.2)	− 0.4% (− 0.8, 0.0)	− 0.6% (− 1.0, − 0.2)	− 0.6% (− 1.0, − 0.2)	− 0.2% (− 0.7, 0.1)
		2 pollutant (+ ozone)	*1.0% (0.4, 1.5)*	0.4% (− 0.1, 0.8)	− 0.2% (− 0.6, 0.2)	− 0.4% (− 0.8, 0.1)	− 0.6% (− 1.0, − 0.2)	− 0.6% (− 1.0, − 0.2)	− 0.2% (− 0.7, 0.2)
		2 pollutant (+ PM2.5)	*1.5% (0.7, 2.2)*	0.3% (− 0.1, 0.8)	− 0.1% (− 0.6, 0.4)	− 0.3% (− 0.8, 0.1)	− 0.4% (− 0.8, 0.0)	− 0.4% (− 0.8, 0..0)	− 0.2% (− 0.7, 0.2)
		3 pollutant (+ ozone & PM2.5)	*1.3% (0.4, 2.1)*	0.3% (− 0.3, 0.8)	− 0.1% (− 0.6, 0.4)	− 0.4% (− 1.0, 0.1)	− 0.4% (− 0.9, 0.0)	− 0.3% (− 0.8, 0.3)	0.0% (− 0.6, 0.5)
PM2.5	warm	1 pollutant	− 0.6% (− 1.2, 0.0)	− 0.1% (− 0.7, 0.5)	− 0.1% (− 0.7, 0.6)	− 0.2% (− 0.7, 0.4)	− 0.5% (− 1.0, 0.0)	− 0.4% (− 1.0, 0.2)	0.1% (− 0.5, 0.7)
		2 pollutant (+ NO_2_)	− 1.4% (− 2.1, − 0.7)	0.1% (− 0.6, 0.7)	0.0% (− 0.7, 0.7)	0.0% (− 0.5, 0.6)	− 0.3% (− 0.9, 0.3)	− 0.4% (− 1.1, 0.2)	0.1% (− 0.5, 0.7)
		2 pollutant (+ ozone)	− 0.1% (− 0.8, 0.6)	− 0.3% (− 1.1, 0.5)	0.1% (− 0.6, 0.8)	0.3% (− 0.3, 1.0)	− 0.6% (− 1.2, 0.1)	− 0.3% (− 1.1, 0.5)	0.0% (− 0.7, 0.8)
		3 pollutant (+ ozone & NO_2_)	− 0.9% (− 1.7, − 0.2)	− 0.1% (− 0.9, 0.7)	0.2% (− 0.5, 0.9)	0.6% (− 0.2, 1.3)	− 0.3% (− 1.1, 0.4)	− 0.3% (− 1.2, 0.5)	− 0.1% (− 0.9, 0.7)
	cold	1 pollutant	0.6% (− 0.2, 1.4)	0.7% (− 0.1, 1.5)	− 0.4% (− 1.1, 0.4)	− 0.4% (− 1.1, 0.3)	− 0.8% (− 1.5, 0.0)	− 0.7% (− 1.4, 0.0)	− 0.1% (− 0.8, 0.5)
		2 pollutant (+ NO_2_)	− 0.5% (− 1.6, 0.6)	0.2% (− 0.8, 1.2)	− 0.2% (− 1.2, 0.8)	0.2% (− 0.8, 1.1)	− 0.3% (− 1.3, 0.9)	− 0.1% (− 1.1, 0.8)	− 0.1% (− 1.0, 0.8)
		2 pollutant (+ ozone)	− 0.2% (− 1.1, 0.7)	0.4% (− 0.4, 1.5)	− 0.4% (− 1.2, 0.5)	− 0.1% (− 0.9, 0.7)	− 0.7% (− 1.5, 0.1)	− 0.4% (− 1.0, 0.5)	0.2% (− 0.5, 1.0)
		3 pollutant (+ ozone & NO_2_)	− 0.8% (− 1.9, 0.4)	0.1% (− 0.9, 1.3)	− 0.2% (− 1.2, 0.8)	0.2% (− 0.7, 1.2)	− 0.3% (− 1.4, 0.8)	− 0.1% (− 1.1, 0.9)	0.0% (− 0.9, 0.9)
	year-round	1 pollutant	0.1% (− 0.3, 0.6)	0.2% (− 0.3, 0.6)	− 0.2% (− 0.7, 0.3)	− 0.3% (− 0.7, 0.1)	− 0.6% (− 1.0, − 0.3)	− 0.6% (− 1.1, − 0.2)	− 0.2% (− 0.6, 0.2)
		2 pollutant (+ NO_2_)	− 0.9% (− 1.5, − 0.3)	0.0% (− 0.5, 0.4)	− 0.2% (− 0.8, 0.4)	− 0.1% (− 0.5, 0.3)	− 0.4% (− 0.9, 0.0)	− 0.4% (− 0.9, 0.1)	− 0.1% (− 0.5, 0.4)
		2 pollutant (+ ozone)	**0.5% (0.0, 0.9**)	0.3% (− 0.3, 0.8)	− 0.1% (− 0.6, 0.4)	− 0.1% (− 0.5, 0.3)	− 0.6% (− 1.0, − 0.2)	− 0.8% (− 1.2, − 0.3)	− 0.3% (− 0.7, 0.1)
		3 pollutant (+ ozone & NO_2_)	− 0.5% (− 1.2, 0.2)	0.1% (− 0.5, 0.7)	0.0% (− 0.6, 0.5)	0.2% (− 0.3, 0.7)	− 0.3% (− 0.8, 0.2)	− 0.6% (− 1.2, 0.0)	− 0.3% (− 0.8, 0.2)

^a^Warm (April to September); cold (October to March); year-round (January to December)

^b^Values in bold indicate significant risk estimates

^c^Values in italics indicates significant risk estimates for all 1-, 2-, and 3-pollutant models

Year-round, circulatory hospitalization risk was also significantly associated with 6-day lagged ozone, 0.2% (0.0, 0.5), with similar associations present in the 2-pollutant model with PM_2.5_ and the 3-pollutant model, 0.3% (0.0, 0.5). The 2-pollutant ozone model adjusted for NO_2_ also estimated a similar, however insignificant, risk with circulatory hospitalization, 0.2% (− 0.0, 0.4). Slightly higher risks of circulatory hospitalization were indicated in single- and multi-pollutant models during the cold season than the warm season when ozone is lagged by 3 and 5 days.

**Fig. 2:**
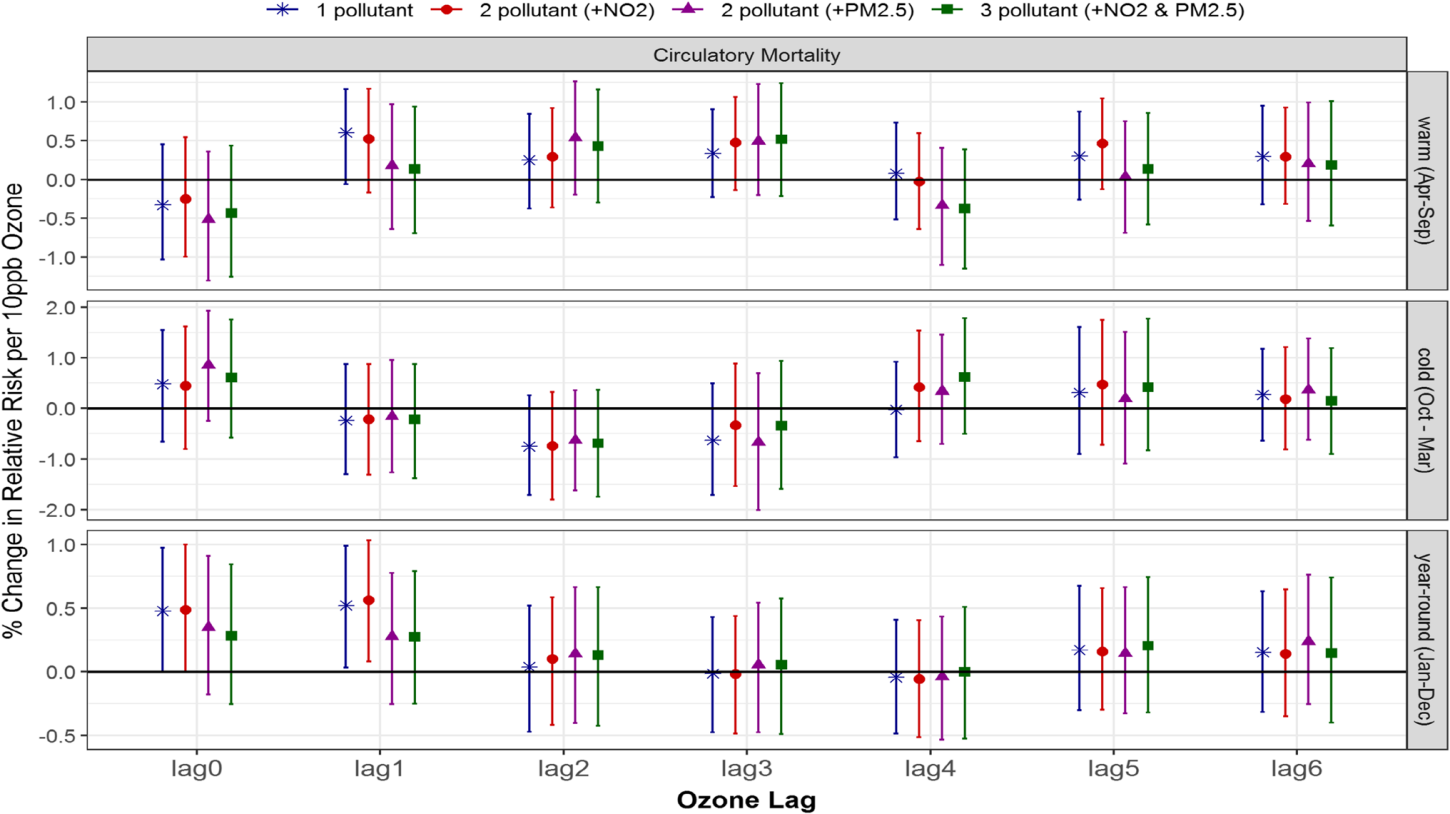
Comparison of estimated associations with 95% credible intervals between ozone and circulatory mortality from multi-pollutant models by season and lag: (*) 1-pollutant model, (●) 2-pollutant model, (▲) another 2-pollutant model, and (■) 3-pollutant model; 3 seasons of warm (Apr to Sept), cold (Oct to Mar) and year-round (Jan to Dec); and (c) 7 lags of 0- to 6-day lagged air pollutant

Unlike circulatory hospitalization, there were no significant associations between ozone and circulatory mortality in the warm or cold season for single-pollutant models, which remained insignificant after adjustment with NO_2_ and/or PM_2.5_ in multi-pollutant models (Fig. 2). However, year-round 1-day lagged ozone was significantly associated with circulatory mortality in the single-pollutant model, 0.5% (0.0, 1.0), and similarly when adjusted for NO_2_ in the 2-pollutant model, 0.6% (0.1, 1.0) (Table 3). In contrast, the risk estimates changed to insignificant, 0.3% (− 0.3, 0.8), with the addition of PM_2.5_ in the 2- and 3-pollutant models.

**Table 3:** % change in risk of circulatory mortality per 10 unit increase in air pollutant concentration

	Season^a^	Model	Air pollutant day lay						
			Lag 0	Lag 1	Lag 2	Lag 3	Lag 4	Lag 5	Lag 6
Ozone	Warm	1 pollutant	− 0.3% (− 1.0, 0.5)	0.6% (− 0.1, 1.2)	0.3% (− 0.4, 0.8)	0.3% (− 0.4, 0.8)	0.1% (− 0.5, 0.7)	0.3% (− 0.3, 0.9)	0.3% (− 0.3, 1.0)
		2 pollutant (+ NO_2_)	− 0.3% (− 1.0, 0.5)	0.5% (− 0.2, 1.2)	0.3% (− 0.4, 0.9)	0.3% (− 0.4, 0.9)	0.0% (− 0.6, 0.6)	0.5% (− 0.1, 1.0)	0.3% (− 0.3, 0.9)
		2 pollutant (+ PM2.5)	− 0.5% (− 1.3, 0.4)	0.2% (− 0.6, 1.0)	0.5% (− 0.2, 1.3)	0.5% (− 0.2, 1.3)	− 0.3% (− 1.1, 0.4)	0.0% (− 0.7, 0.8)	0.2% (− 0.5, 1.0)
		3 pollutant (+ NO_2_ & PM2.5)	− 0.4% (− 1.3, 0.4)	0.1% (− 0.7, 0.9)	0.4% (− 0.3, 1.2)	0.4% (− 0.3, 1.2)	− 0.4% (− 1.1, 0.4)	0.1% (− 0.6, 0.9)	0.2% (− 0.6, 1.0)
	Cold	1 pollutant	0.5% (− 0.7, 1.5)	− 0.2% (− 1.3, 0.9)	− 0.8% (− 1.7, 0.3)	− 0.8% (− 1.7, 0.3)	0.0% (− 1.0, 0.9)	0.3% (− 0.9, 1.6)	0.3% (− 0.6, 1.2)
		2 pollutant (+ NO_2_)	0.4% (− 0.8, 1.6)	− 0.2% (− 1.3, 0.9)	− 0.7% (− 1.8, 0.3)	− 0.7% (− 1.8, 0.3)	0.4% (− 0.7, 1.5)	0.5% (− 0.7, 1.7)	0.2% (− 0.8, 1.2)
		2 pollutant (+ PM2.5)	0.9% (− 0.3, 1.9)	− 0.2% (− 1.3, 1.0)	− 0.6% (− 1.6, 0.4)	− 0.6% (− 1.6, 0.4)	0.3% (− 0.7, 1.5)	0.2% (− 1.1, 1.5)	0.4% (− 0.6, 1.4)
		3 pollutant (+ NO_2_ & PM2.5)	0.6% (− 0.6, 1.8)	− 0.2% (− 1.4, 0.9)	− 0.7% (− 1.7, 0.4)	− 0.7% (− 1.7, 0.4)	0.6% (− 0.5, 1.8)	0.4% (− 0.8, 1.8)	0.1% (− 0.9, 1.2)
	Year-round	1 pollutant	0.5% (− 0.0, 1.0)	**0.5% (0.0, 1.0)**^**b**^	0.0% (− 0.5, 0.5)	0.0% (− 0.5, 0.5)	0.0% (− 0.5, 0.4)	0.2% (− 0.3, 0.7)	0.2% (− 0.3, 0.6)
		2 pollutant (+ NO_2_)	0.5% (− 0.0, 1.0)	**0.6% (0.1, 1.0)**	0.1% (− 0.4, 0.6)	0.1% (− 0.4, 0.6)	− 0.1% (− 0.5, 0.4)	0.2% (− 0.3, 0.7)	0.1% (− 0.3, 0.6)
		2 pollutant (+ PM2.5)	0.3% (− 0.2, 0.9)	0.3% (− 0.3, 0.8)	0.1% (− 0.4, 0.7)	0.1% (− 0.4, 0.7)	0.0% (− 0.5, 0.4)	0.1% (− 0.3, 0.7)	0.2% (− 0.3, 0.8)
		3 pollutant (+ NO_2_ & PM2.5)	0.3% (− 0.3, 0.8)	0.3% (− 0.3, 0.8)	0.1% (− 0.4, 0.7)	0.1% (− 0.4, 0.7)	0.0% (− 0.5, 0.5)	0.2% (− 0.3, 0.7)	0.1% (− 0.4, 0.7)
NO_2_	Warm	1 pollutant	− 0.4% (− 2.2, 1.4)	0.1% (− 1.5, 1.7)	− 0.2% (− 2.0, 1.5)	− 1.6% (− 3.3, 0.0)	0.4% (− 1.1, 1.9)	− 0.2% (− 1.8, 1.5)	0.3% (− 2.1, 2.8)
		2 pollutant (+ ozone)	− 0.2% (− 2.0, 1.6)	0.1% (− 1.6, 1.9)	− 0.4% (− 2.1, 1.3)	− 2.0% (− 3.9, − 0.4)	0.3% (− 1.2, 1.8)	− 0.4% (− 2.1, 1.4)	− 0.4% (− 2.4, 1.9)
		2 pollutant (+ PM2.5)	− 0.3% (− 2.1, 1.5)	− 0.2% (− 2.0, 1.7)	− 0.1% (− 1.9, 1.9)	− 1.9% (− 3.8, − 0.2)	0.2% (− 1.4, 1.9)	− 0.8% (− 2.5, 1.0)	− 0.3% (− 2.6, 2.1)
		3 pollutant (+ ozone & PM2.5)	− 0.6% (− 2.6, 1.6)	− 0.1% (− 1.7, 1.8)	0.1% (− 2.4, 2.4)	− 2.5% (− 4.5, − 0.7)	0.1% (− 1.5, 1.6)	− 0.8% (− 2.5, 1.1)	− 0.1% (− 3.1, 2.8)
	Cold	1 pollutant	− 0.6% (− 1.9, 0.6)	0.5% (− 0.6, 1.6)	0.6% (− 0.6, 1.8)	1.0% (− 0.1, 2.2)	1.0% (− 0.1, 2.0)	0.3% (− 0.7, 1.3)	− 0.6% (− 1.6, 0.4)
		2 pollutant (+ ozone)	− 0.2% (− 1.7, 1.1)	0.3% (− 0.9, 1.5)	0.3% (− 0.9, 1.6)	0.8% (− 0.4, 2.0)	1.1% (− 0.1, 2.3)	0.7% (− 0.4, 1.7)	− 0.6% (− 1.7, 0.5)
		2 pollutant (+ PM2.5)	− 1.2% (− 2.6, 0.2)	− 0.2% (− 1.6, 1.1)	0.0% (− 1.4, 1.4)	1.2% (− 0.3, 2.7)	1.0% (− 0.3, 2.3)	0.8% (− 0.5, 2.1)	− 0.9% (− 2.2, 0.4)
		3 pollutant (+ ozone & PM2.5)	− 0.7% (− 2.7, 1.2)	− 0.5% (− 2.3, 1.4)	− 0.9% (− 2.7, 1.1)	0.8% (− 1.0, 2.7)	0.9% − 0.9, 2.7)	1.1% (− 0.7, 2.9)	− 1.0% (− 2.8, 0.7)
	Year-round	1 pollutant	− 0.2% (− 1.2, 0.7)	0.6% (− 0.3, 1.5)	0.3% (− 0.4, 1.1)	0.0% (− 0.7, 0.8)	0.4% (− 0.4, 1.2)	− 0.2% (− 1.0, 0.7)	− 0.8% (− 1.5, 0.0)
		2 pollutant (+ ozone)	0.0% (− 0.9, 1.0)	0.8% (− 0.1, 1.7)	0.4% (− 0.4, 1.2)	− 0.1% (− 0.8, 0.7)	0.4% (− 0.4, 1.2)	− 0.1% (− 0.9, 0.8)	− 0.9% (− 1.6, − 0.1)
		2 pollutant (+ PM2.5)	− 0.6% (− 1.5, 0.3)	0.0% (− 1.0, 1.1)	0.1% (− 0.8, 1.0)	0.1% (− 0.8, 1.0)	0.5% (− 0.4, 1.3)	0% (− 0.9, 0.9)	− 0.7% (− 1.6, 0.1)
		3 pollutant (+ ozone & PM2.5)	− 0.4% (− 1.5, 0.7)	0.1% (− 1.0, 1.2)	0.1% (− 0.9, 1.1)	0.0% (− 1.0, 1.0)	0.3% (− 0.7, 1.3)	0.1% (− 0.9, 1.2)	− 0.8% (− 1.8, 0.3)
PM2.5	Warm	1 pollutant	0.2% (− 1.2, 1.5)	1.1% (− 0.1, 2.3)	− 0.2% (− 1.6, 1.0)	− 0.2% (− 1.4, 0.9)	0.6% (− 0.6, 1.8)	1.1% (− 0.2, 2.6)	0.4% (− 0.7, 1.5)
		2 pollutant (+ NO_2_)	0.2% (− 1.2, 1.6)	1.2% (− 0.2, 2.4)	− 0.1% (− 1.6, 1.2)	0.3% (− 0.9, 1.5)	0.6% (− 0.6, 1.9)	**1.4% (0.1, 2.8)**	0.7% (− 0.5, 1.8)
		2 pollutant (+ ozone)	0.5% (− 1.0, 2.0)	1.0% (− 0.6, 2.3)	− 0.5% (− 2.1, 0.9)	− 0.8% (− 2.3, 0.6)	1.0% (− 0.4, 2.4)	1.1% (− 0.4, 2.9)	0.0% (− 1.3, 1.3)
		3 pollutant (+ ozone & NO_2_)	0.6% (− 0.9, 2.1)	1.1% (− 0.5, 2.5)	− 0.5% (− 2.1, 1.0)	− 0.2% (− 1.7, 1.3)	1.0% (− 0.5, 2.7)	1.3% (− 0.2, 3.1)	0.4% (− 1.1, 1.9)
	Cold	1 pollutant	0.2% (− 2.0, 2.4)	1.3% (− 0.3, 3.1)	1.1% (− 0.4, 2.6)	0.5% (− 1.2, 2.5)	0.6% (− 1.6, 2.6)	− 0.2% (− 2.0, 1.3)	− 0.2% (− 1.7, 1.3)
		2 pollutant (+ NO_2_)	1.4% (− 0.9, 3.6)	1.5% (− 0.5, 3.5)	1.5% (− 0.5, 3.3)	− 0.5% (− 2.9, 1.9)	0.0% (− 2.5, 2.4)	− 1.0% (− 3.0, 0.9)	0.6% (− 1.4, 2.5)
		2 pollutant (+ ozone)	1.0% (− 1.3, 3.1)	1.1% (− 0.5, 2.9)	0.8% (− 0.7, 2.3)	0.2% (− 1.9, 2.4)	0.8% (− 1.5, 3.0)	− 0.1% (− 1.8, 1.5)	− 0.1% (− 1.7, 1.4)
		3 pollutant (+ ozone & NO_2_)	1.7% (− 0.5, 3.9)	1.5% (− 0.6, 3.5)	1.4% (− 0.5, 3.3)	− 0.6% (− 3.1, 1.9)	0.1% (− 2.5, 2.6)	− 1.0% (− 3.2, 1.0)	0.5% (− 1.4, 2.4)
	Year-round	1 pollutant	0.8% (− 0.3, 1.9)	*1.4% (0.4, 2.2)*^*c*^	0.1% (− 0.9, 1.0)	− 0.3% (− 1.2, 0.5)	0.2% (− 0.8, 1.3)	0.0% (− 1.0, 1.1)	− 0.4% (− 1.2, 0.4)
		2 pollutant (+ NO_2_)	**1.2% (0.1, 2.2)**	*1.3% (0.3, 2.3)*	0.0% (− 1.2, 1.1)	− 0.4% (− 1.4, 0.5)	0.0% (− 1.1, 1.3)	0.0% (− 1.0, 1.2)	0.1% (− 0.9, 1.0)
		2 pollutant (+ ozone)	0.8% (− 0.4, 1.9)	*1.3% (0.3, 2.2)*	0.2% (− 0.9, 1.1)	− 0.4% (− 1.3, 0.6)	0.3% (− 0.8, 1.4)	− 0.1% (− 1.1, 1.1)	− 0.7% (− 1.6, 0.1)
		3 pollutant (+ ozone & NO_2_)	1.1% (− 0.0, 2.1)	*1.2% (0.1, 2.2)*	0.0% (− 1.2, 1.2)	− 0.5% (− 1.6, 0.7)	0.1% (− 1.1, 1.5)	− 0.1% (− 1.3, 1.1)	− 0.2% (− 1.3, 0.8)

^a^Warm (April to September); cold (October to March); year-round (January to December)

^b^Values in bold indicates significant risk estimates

^c^Values in italics indicates significant risk estimates for all 1-, 2-, and 3-pollutant models

### Multi-pollutant associations between NO_2_ and circulatory hospitalization and mortality

**Fig. 3:**
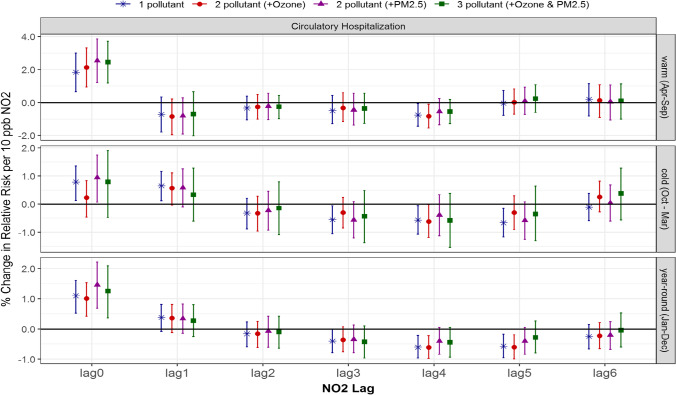
Comparison of estimated associations with 95% credible intervals between NO2 and circulatory hospitalization from multi-pollutant models by season and lag: (*) 1-pollutant model, (●) 2-pollutant model, (▲) another 2-pollutant model, and (■) 3-pollutant model; 3 seasons of warm (Apr to Sept), cold (Oct to Mar) and year-round (Jan to Dec); and (c) 7 lags of 0- to 6-day lagged air pollutant

Overall, the associations between NO_2_ and circulatory hospitalization were highest for same-day exposure, or in the cold season up to 1-day lagged NO_2_, after which the associations declined and remained close to null. As with ozone, some seasonal differences were present in the risk of circulatory hospitalization associated with NO_2_; however, the significant associations were more consistent in the warm and year-round seasons compared to cold season (Fig. 3). In the warm season, 0-day lagged NO_2_ returned significant associations in the single-pollutant model, 1.8% (0.7, 3.0), and slightly stronger in the 2-pollutant models, 2.1% (0.9, 3.3) and 2.5% (1.2, 3.9) when adjusted for ozone and PM_2.5_, respectively, and the 3-pollutant model, 2.5% (1.2, 3.7). In the cold season, 0-day lagged NO_2_ returned a significant association of 0.8% (0.1, 1.4), and remained so when adjusted for PM_2.5_, 0.9% (0.1, 1.7), but became insignificant when adjusted for ozone in both the 2- and 3-pollutant models. In contrast, 1-day lagged NO_2_ and circulatory hospitalization were significantly associated in the single-pollutant model only. Year-round the risk estimates were lower than the warm season, but still significant independent of the model composition.

**Fig. 4:**
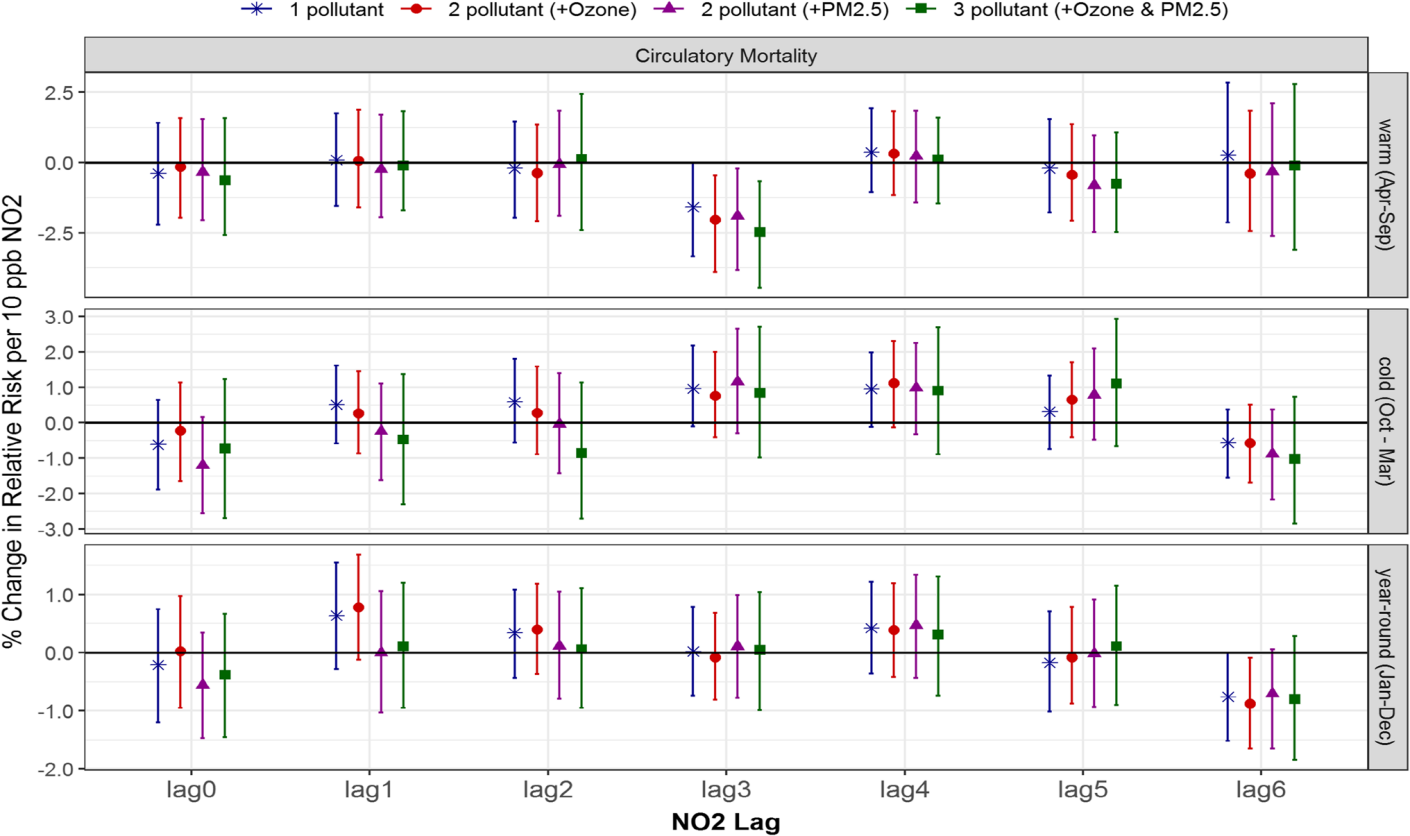
Comparison of estimated associations with 95% credible intervals between NO2 and circulatory mortality from multi-pollutant models by season and lag: (*) 1-pollutant model, (●) 2-pollutant model, (▲) another 2-pollutant model, and (■) 3-pollutant model; 3 seasons of warm (Apr to Sept), cold (Oct to Mar) and year-round (Jan to Dec); and (c) 7 lags of 0- to 6-day lagged air pollutant

There were no significant positive associations between NO_2_ and circulatory mortality. For seasonal differences, 3-day lagged NO_2_ showed somewhat higher associations during the cold season than the warm season, particularly for the 1- and 2-pollutant models (Fig. 4).

### Multi-pollutant associations between PM_2.5_ and circulatory hospitalization and mortality

**Fig. 5:**
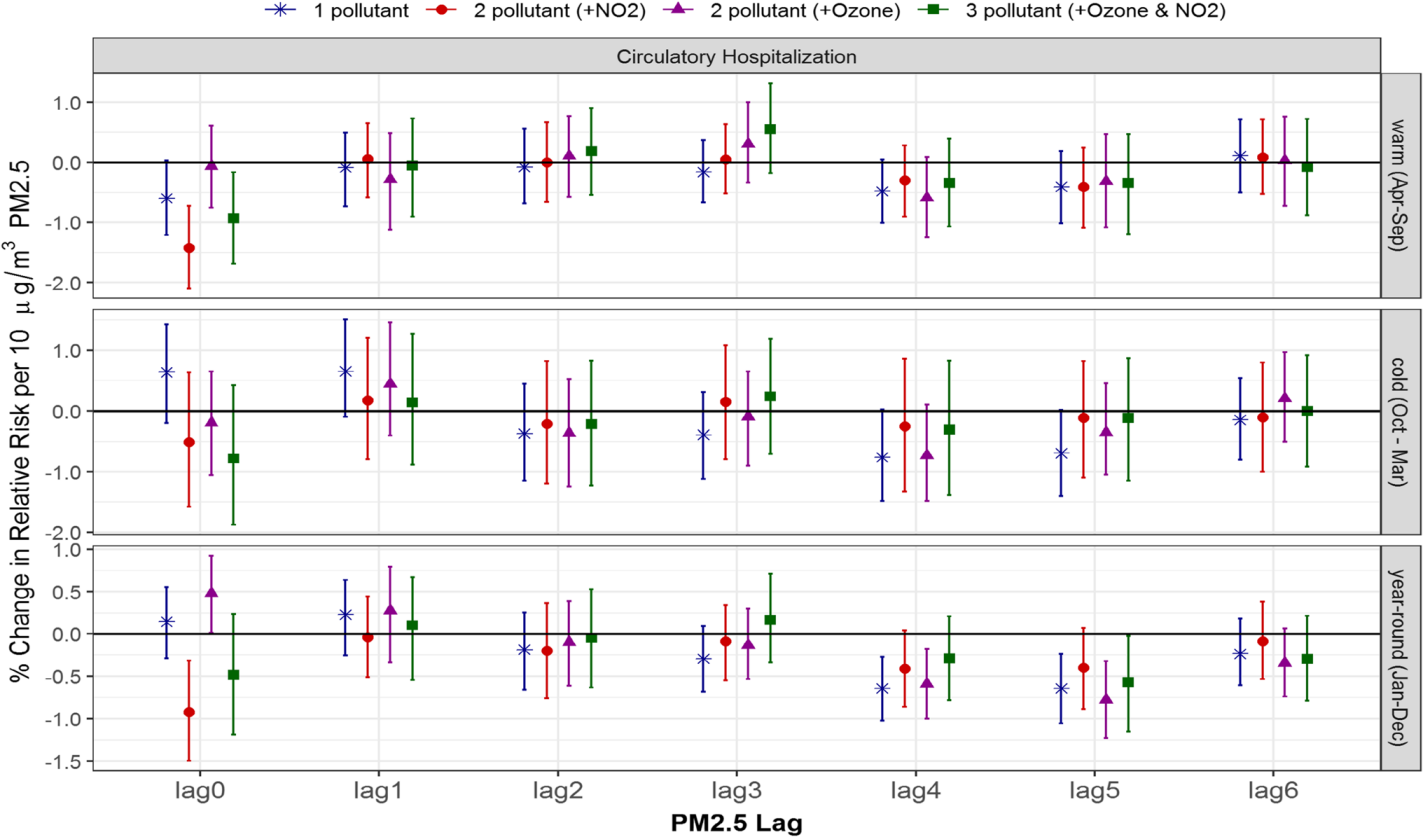
Comparison of estimated associations with 95% credible intervals between PM2.5 and circulatory hospitalization from multi-pollutant models by season and lag: (*) 1-pollutant model, (●) 2-pollutant model, (▲) another 2-pollutant model, and (■) 3-pollutant model; 3 seasons of warm (Apr to Sept), cold (Oct to Mar) and year-round (Jan to Dec); and (c) 7 lags of 0- to 6-day lagged air pollutant

Unlike ozone and NO_2_, no significant positive associations were present between PM_2.5_ and circulatory hospitalization in single-pollutant models (Fig. 5). When adjusted for ozone, year-round 0-day lagged PM_2.5_ did have a significant association, 0.5% (0.0, 0.9); however, this became insignificant with the addition of NO_2_ in the 3-pollutant model, − 0.5% (− 1.2, 0.2), or when adjusted for just NO_2_, − 0.9% (− 1.5, − 0.3). The associations between PM_2.5_ and circulatory hospitalization also exhibited more pronounced differences depending on other pollutants, lag days and season.

**Fig. 6:**
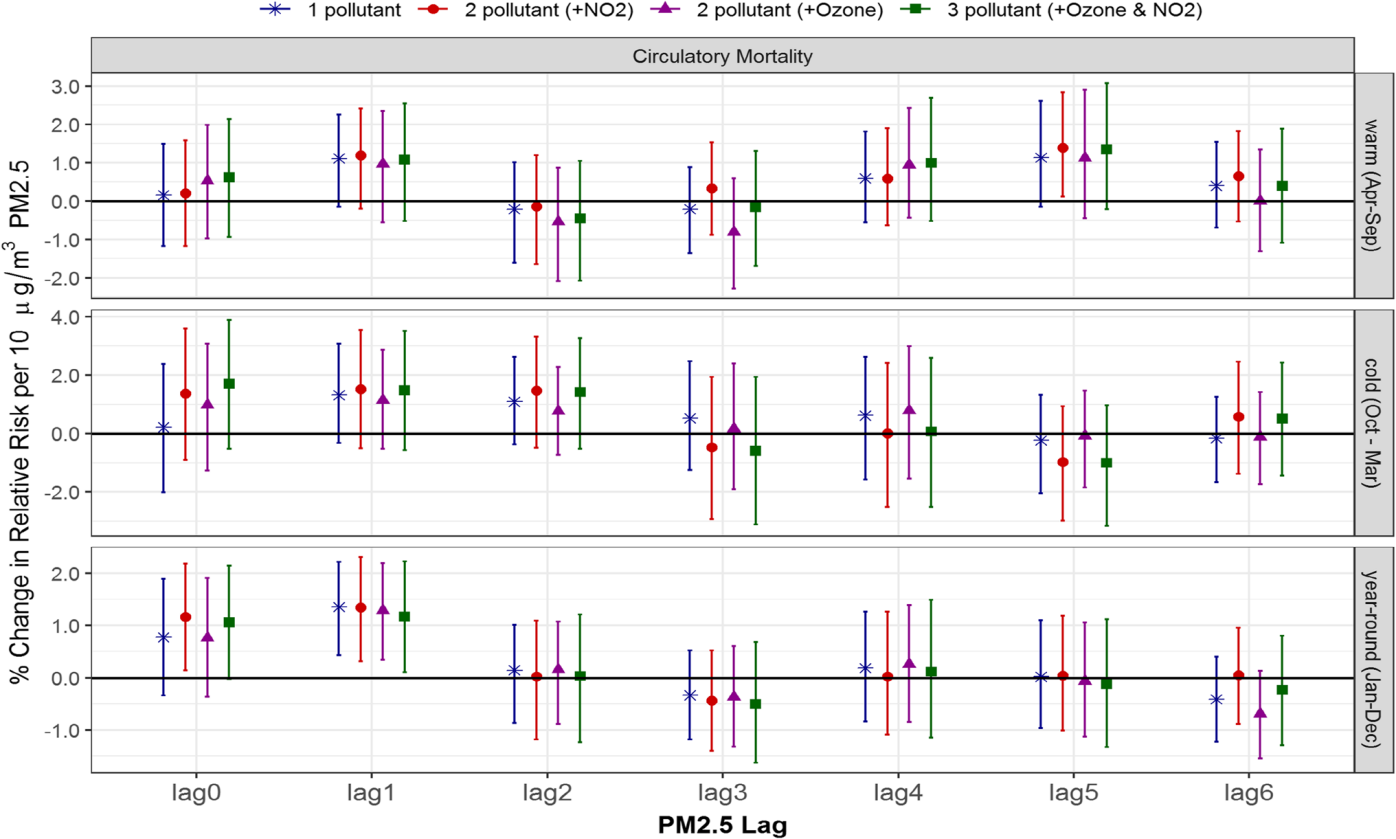
Comparison of estimated associations with 95% credible intervals between PM2.5 and circulatory mortality from multi-pollutant models by season and lag: (*) 1-pollutant model, (●) 2-pollutant model, (▲) another 2-pollutant model, and (■) 3-pollutant model; 3 seasons of warm (Apr to Sept), cold (Oct to Mar) and year-round (Jan to Dec); and (c) 7 lags of 0- to 6-day lagged air pollutant

In single- and multi-pollutant models with 1-day lagged air pollutants, PM_2.5_ was significantly and consistently associated with circulatory mortality year round (Fig. 6): 1–pollutant model, 1.4% (0.4, 2.2); 2-pollutant models adjusted for NO_2_, 1.3% (0.3, 2.3), and ozone, 1.3% (0.3, 2.2); and the 3-pollutant model, 1.2% (0.1, 2.2). In contrast, PM_2.5_ adjusted for NO_2_ only in the 2-pollutant model was significantly associated with circulatory mortality: 0-day lagged PM_2.5_ year-round, 1.2% (0.1, 2.2), and 5-day lagged PM_2.5_ in the warm season, 1.4% (0.1, 2.8). In both cases, all other models (with or without ozone) yielded insignificant results.

## Discussion

We investigated circulatory hospitalizations and mortality attributable to short-term exposure to ambient air pollutants such as ozone, NO_2_, and PM_2.5_ through multi-pollutant models with the same lag structure within a week time window, using lags of 0 to 6 days, and further examined seasonal adjustment of other pollutants through 2- and 3-pollutant models. We found a few seasonal differences in the adjusted effects of other air pollutants. For example, significant effect of NO_2_ on circulatory hospitalizations remained with adjustment of ozone and PM_2.5_ during warm season but became insignificant with adjustment of ozone during cold season. Contrary to expectation, we found overall little additive or antagonistic risk of circulatory health outcomes from the three air pollutants. This is similar to the result of respiratory health outcomes from the same air pollutants (Parajuli et al. 2021). This could be explained in part by relatively low-to-moderate correlations between the specified air pollutants.

Hourly daytime ozone and NO_2_ are generally expected to be negatively correlated since nitrogen oxides (NO_x_) utilize ozone during the morning, but fuel the photochemical buildup of ozone later in the day. Averaging pollutant concentrations over longer periods of time obscures this relationship; 24 h NO_2_ concentrations will depend heavily on local emissions and the degree of atmospheric mixing, whereas 8-h max ozone is influenced by the presence of sunlight to facilitate the photochemical reaction. Averaging concentrations from multiple stations further masks the relationship as the ozone-NO_2_ dynamic varies depending on the surrounding level of development and traffic.

Warm-season PM_2.5_ and ozone are both influenced by meteorological conditions such as temperature inversions which reduce atmospheric mixing, and long-range transport from more polluted areas. Warm, stable meteorological conditions accumulate PM_2.5_ and have less cloud cover, allowing for ozone formation. Ozone concentrations decline significantly during the cold-season, in contrast to PM_2.5_ and NO_x_ which are still emitted and can accumulate together under appropriate meteorological conditions.

While ozone and NO_2_ had low correlations regardless of season, PM_2.5_ had seasonal high correlations with ozone for warm season in Ontario only (Table S2), and with NO_2_ for cold season in Quebec (Table S2). These localized high correlations resulted in overall low to moderate correlations (between ± 0.5) nationally (Figure S2). The national low-to-moderate correlation might have resulted in little change in risk estimates from single- to multi-pollutant models, which needs further discussion.

We may expect seasons with higher air pollutant concentrations would result in higher risk of circulatory diseases, however this study counterintuitively reported the opposite. For example, ozone’s effect was stronger during the cold season, whereas its concentrations were higher in the warm season. Similarly, NO_2_’s effect was stronger in the warm season but its concentrations were higher in the cold season. Both examples can be explained in two ways: first, the reported effect (or association) represents the increase (% change) in the circulatory health outcomes per 10 unit increase in concentrations, which should be multiplied by the concentrations for the total effect. Therefore, higher concentrations of ozone during the warm season could result in a higher total effect of ozone even though its per-unit-effect was weaker than in the cold season. Second, we estimated the effect of air pollution through short-term exposures (e.g., within a week) in model [1]. The effects are the associations between daily variations in both air pollution concentrations and counts of health outcomes. Therefore, the effect would be highest when the daily count of health outcomes is synchronized (up/down changes) with daily pollutant concentrations. Such synchronization in daily variations can occur independent of air pollution concentration levels.

### Changes in effect of ozone with adjustment of NO_2_ and PM_2.5_

Here, we use the term “change” to refer to a difference in statistical significance of the two associations estimated by single- vs. multi-pollutant models: for example, a significant association from a single-pollutant model was changed to an insignificant association from a multi-pollutant model, or vice versa. We do not intend for the term “change” to imply a statistically significant difference between the two estimated associations.

For the adverse health effect of ozone, based on low correlation with NO_2_ (± 0.2 on average) and moderate correlation with PM_2.5_ (0.5 on average for the warm season), we anticipated no change in association with circulatory health outcomes with adjustment of NO_2_ but some change with adjustment of PM_2.5_ for warm season. However, there was no evidence of a warm-season adjusted effect of PM_2.5_ on the association between ozone and circulatory hospitalization (Table 2) and mortality (Table 3).

While there was no change in the effect of ozone after accounting for NO_2_ and/or PM_2.5_ during the warm season, a few changes were observed during the cold season and year-round. For circulatory hospitalization, a cold-season significant association of 0.6% (0.1, 1.0) for 3-day lagged ozone changed to insignificant in the 2-pollutant (with NO_2_ and PM_2.5_, respectively) and 3-pollutant (with NO_2_ and PM_2.5_ together) models. A year-round significant association of 0.2% (0.0, 0.5) for 3-day lagged ozone became insignificant after adjusting for NO_2_ but remained significant after adjusting for PM_2.5_ [0.3% (0.0, 0.5)] and for both PM_2.5_ and NO_2_ [0.3% (0.0, 0.5)]. For circulatory mortality, a year-round significant association of 0.5% (0.0, 1.0) for 1-day lagged ozone remained stable after adjusting for NO_2_ [0.6% (0.1, 1.0)] but became insignificant after adjusting for PM_2.5_ with/without NO_2_. This was expected for the warm season but observed for year-round estimates instead.

In contrast with the study finding, a study in the US reported that significant positive associations remained between ozone and circulatory mortality adjusted for PM_2.5_ [hazard risk of 1.03 (1.01–1.05)] that were unchanged with further adjustment for NO_2_ (Turner et al. 2016). Yet consistent effect of further adjustment for other pollutants in our study is not matched with other studies. For example, the positive effect of ozone on circulatory outpatient visits, 2.83% (0.65, 5.06), increased after adjusting for NO_2_ in Fuzhou, China (Jiang et al. 2020), when the concentration of ozone was higher than 100 μg/L. However, this study did not report the correlation between ozone and NO_2_, and it is unclear if the increased effect of ozone with NO_2_ was linked to their correlation.

Taken together, changes in the effect of ozone after accounting for NO_2_ and/or PM_2.5_ were not explained by their seasonal correlations solely. Overall, the magnitude of the associations (the 95% credible interval) from 1-, 2-, to 3-pollutant models were quite comparable (Figs. 1 and 2), and this implies that single-pollutant models for ozone without adjustment of NO_2_ and/or PM_2.5_ did not result in under- or over-estimate.

### Changes in effect of NO_2_ with adjustment of ozone and PM_2.5_

For the adverse health effect of NO_2_, based on low correlation with ozone (± 0.2 on average) and moderate correlation with PM_2.5_ (0.5 on average for cold season), we anticipated no change in association with circulatory health outcomes with adjustment of ozone but some change after adjusting for PM_2.5_ for cold season. As expected, there was no evidence of a warm-season adjusted effect of ozone and/or PM_2.5_ on the association between NO_2_ and circulatory hospitalization (Table 2) and mortality (Table 3).

Unlike the warm season, a few changes were observed during the cold season. For circulatory hospitalization (not mortality), a cold-season significant association of 0.8% (0.1, 1.4) for no lagged NO_2_ changed to insignificant after adjusting for ozone but remained significant with PM_2.5_. This is contrary to what is expected, and thus cannot be explained by their seasonal correlations solely. However, the 95% credible intervals from 1-, 2-, to 3-pollutant models were quite comparable (Figs. 3 and 4), which implies that overall the single-pollutant model for NO_2_ without adjustment of ozone and/or PM_2.5_ did not result in under- or over-estimates.

Similar to the consistent effect of NO_2_ for other pollutants during the warm season in our study, a Brazilian study reported that NO_2_ effect remained significant in multi-pollutant (ozone and others) models for circulatory mortality among the elderly living in São Paulo between 2000 and 2011 (Costa et al. 2017). However, this study did not report correlations and seasonal differences.

### Changes in effect of PM_2.5_ with adjustment of ozone and NO_2_

For the adverse health effect of PM_2.5_, based on moderate correlation with ozone (0.5; ranged between 0.2 and 0.7 for warm season) and with NO_2_ (0.5; ranged between 0.1 and 0.8 for cold season), we anticipated seasonal changes in association with circulatory health outcomes with adjustment of ozone and/or NO_2_. While there was no evidence of a cold-season adjusted effect of NO_2_ on the association between PM_2.5_ and circulatory hospitalization (Table 2) and mortality (Table 3), a warm-season adjusted effect of NO_2_ (not ozone) was observed in an unexpected way: an insignificant effect of 5-day lagged PM_2.5_ became significant after adjusting for NO_2_ but remained unchanged after adjusting for ozone. This is opposite to what is expected based on their seasonal correlations.

As seen for ozone and NO_2_, the changes in significance may be related to small effect sizes very close to zero. Overall, the 95% credible intervals from 1-, 2-, to 3-pollutant models were quite comparable (Figs. 5 and 6), and thus the single-pollutant model for PM_2.5_ without adjustment of ozone and/or NO_2_ did not result in under- or over-estimates in this study.

Yet, a Taiwan study reported different results on season in two-pollutant models. The effect of PM_2.5_ on circulatory mortality remained significant when ozone was added in the regression model both on warm and cool days in Taipei during 2006–2008 (Cheng et al. 2016). Another study in the USA examined hospital admissions for CVD during 1987–1995 (Moolgavkar 2000) and reported that the effect of the gases (ozone and NO_2_) remained stable, while the effect of PM_2.5_ became unstable and insignificant in 2-pollutant models. This study offered a perspective on the different adjusted effects of other air pollutants in terms of gas vs. non-gas, which warrants further investigation.

### Limitations and strength

Our study has some limitations that warrant discussion. First, this is an ecological study using city level data, not individual level data, and thus misclassification of exposure was unavoidable. This could bring in unmeasurable bias in estimated associations. Second, our statistical model assumed no change in the associations over the study period (2001–2012). The reported associations should be interpreted as 12-year overall estimates, not capturing year-by-year variations. Third, we did not consider interactions among the specified three air pollutants. This is because the range of PM_2.5_ and NO_2_ were quite narrow, which would result in unstable estimates. Fourth, we used the same lag structure for multi-pollutant models, since we focused on adjusted effects of multi-pollutants on the same day. This limited our ability to detect adjusted effects of air pollutants with different lags. Fifth, we did not consider age groups such as seniors vs. non-senior due to low rate of non-senior (< 65 years) hospitalization (37%) and mortality (14%). This may have limited our ability to detect age-related differences in the associations of interest. Finally, we did not account for the possible effects of environmental noise (e.g., nighttime road traffic), which has been shown to be related to symptoms of insomnia (Evandt et al. 2017; Halperin 2014). By way of compromised sleep, an indirect pathway has been recently proposed between environmental noise and adverse cardiovascular outcomes (Gilani and Mir 2021, 2022), which may confound air pollution exposure related health risks.

Our study also has several strengths. We reported associations between three important air pollutants and circulatory health outcomes in the Canadian context for a long period, 12 years between 2001 and 2012, and explored changes in the associations by adjusting for other pollutants. To our knowledge, this is the first recent Canadian study to report health effects of ozone, NO_2_, and PM_2.5_ through multi-pollutant models by season, comparing circulatory hospitalization and mortality in a large number of cities. In addition, our use of ground monitoring data over model-driven estimates is a strength. This likely reduced exposure misclassification in our analyses, since the ground monitoring data (i.e., NAPS data) do not depend on specific models on temporal or spatial variations. Finally, we confirmed that the degree of under- or over-estimates from single-pollutant models were negligible, compared to the multi-pollutant models, which can, in part, be explained by the relatively low correlations among the multi-pollutants.

## Conclusion

The study findings indicate the adjusted effects of short-term exposures to multi-pollutants were inconsistent between circulatory hospitalization and mortality, which cannot be explained solely by correlations among the three common air pollutants (ozone, NO_2_, and PM_2.5_). Overall, we found statistically insignificant differences in risk of circulatory health outcomes between single- and multi-pollutant models, indicating little additive effect from the three specified air pollutants. Although further investigations are required, our study results suggest little under- or over-estimates from 1-pollutant models, compared to 2- and 3-pollutant models in Canada. The inconsistent findings from previous studies and this study indicate the need of comparable study design for multi-pollutant effect analysis.

## Supplementary Information

Below is the link to the electronic supplementary material.

Supplementary file1 (PDF 1028 KB)

## Data Availability

Air pollution and temperature data are open to the public; hospitalization and mortality data for province Quebec are accessible with restrictions through Commission d’accès à l’information du Québec (https://www.cai.gouv.qc.ca/); and hospitalization and mortality data for other provinces are accessible with restrictions through the CIHI (https://www.cihi.ca/en/access-data-and-reports) and Statistics Canada (Canadian Research Data Centre Network, https://crdcn.org/map_transition), respectively.

## Abbreviations

CDs: Census divisions
ICD-10: International Classification of Diseases version 10
NAPS: National Air Pollution Surveillance System
ECCC: Environment and Climate Change Canada
CIHI: Canadian Institute of Health Information
O_3_: Ground-level ozone
NO_x_: Nitrogen oxide
NO_2_: Nitrogen dioxide
PM_2.5_: Particulate matter of diameter of less than 2.5 µm
CVD: Cardiovascular disease
