# Supplementary material for: Circulatory health risks from additive multi-pollutant models: short-term exposure to three common air pollutants in Canada
Source: Environ Sci Pollut Res Int. 2022 Sep 29;30(6):15740–55. doi: 10.1007/s11356-022-22947-4 (PMC9908686; doi:10.1007/s11356-022-22947-4)
Supplement: Supplementary file 1 — Supplementary file1 (PDF 1028 KB) [file 11356_2022_22947_MOESM1_ESM.pdf]

Supplementary Materials

Circulatory Health Risks from Additive Multi-pollutant Models: Short-term Exposure to Three Common Air Pollutants in Canada

Hwashin H. Shin[a,b,*], James Owen[a], Aubrey Maquiling[a], Rajendra P. Parajuli[c], Marc Smith-Doiron[a]

[a] Environmental Health Science and Research Bureau, Health Canada, Ottawa, ON, Canada

[b] Dept. of Mathematics and Statistics, Queen's University, Kingston, ON, Canada

[c] Central Department of Zoology, Central Campus, Institute of Science & Technology (IOST), Tribhuvan University, Kritipur-1, Kathmandu, Nepal

*Corresponding author: Hwashin.Shin@ hc-sc.gc.ca

Note: The order of materials in supplementary follows the order of the main text.

Table of Contents

Figure S1. Canada map on the 24 cities selected for the study……..2
Table S1. City-specific annual average concentrations of Ozone, NO2, PM2.5, and temperature concentrations by season for 2001-2012……….3
Table S2. City-specific annual average Pearson's correlations between Ozone, NO2, and PM2.5 for 2001-2012……..4
Table S3. City-specific annual average of respiratory mortality ratio to population by sex and age (2001-2012)……….5
Figure S2 Annual Pearson correlation between three air pollutants for 2001-2012 by season ….5
Figure S3 Diagram on significance of associations between 3 air pollutants and circulatory hospitalization and mortality, respectively, by model, season and lag ……...6

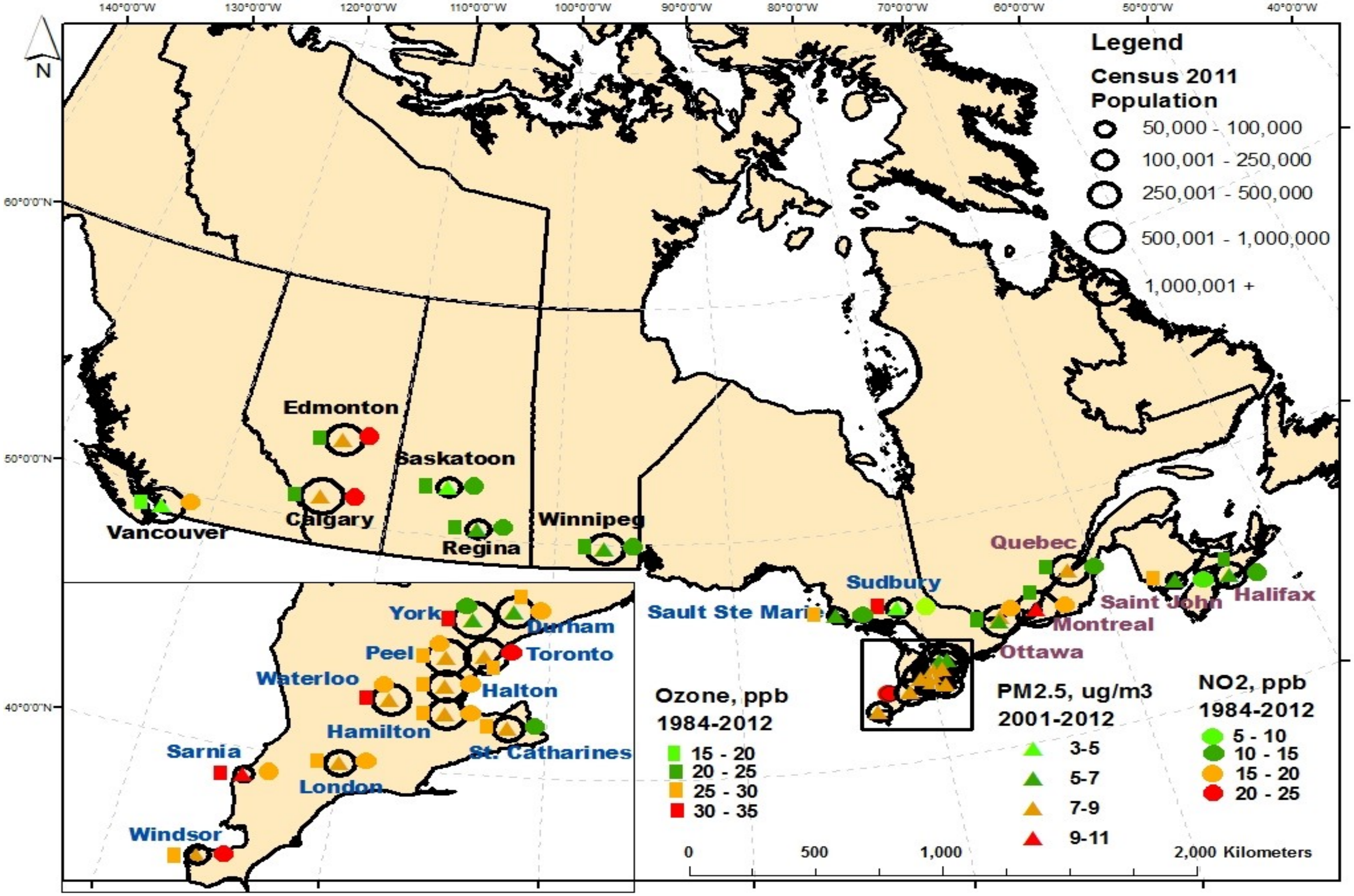

Figure S1: Canada map on the 24 cities selected for the study. (1) empty circle size: population size; (2) shape: solid square (ozone), solid circle (NO2) and solid triangle (PM2.5); and (3) color: concentrations in 4 levels of low to high in green to red

Table S1. City-specific annual average concentrations of Ozone, NO2, PM2.5, and temperature concentrations by season for 2001-2012

City	Ozone[a] in ppb (SD[b])		NO2[c] in ppb (SD)		PM2.5[d] in μg/m^3 (SD)		Temperature[e] in °C (SD)	
	Warm[f]	Cold[f]	Warm[f]	Cold[f]	Warm[f]	Cold[f]	Warm[f]	Cold[f]
Halifax	26 (3.9)	27 (2.7)	12 (3.8)	13 (4.3)	6 (0.9)	4 (1.1)	14 (0.6)	1 (1.0)
Saint John	32 (1.0)	**32** (1.1)	7 (3.0)	6 (2.3)	7 (1.4)	5 (1.0)	12 (0.5)	0 (1.0)
Quebec City	33 (2.4)	26 (1.2)	9 (1.8)	14 (2.9)	8 (0.7)	9 (1.4)	15 (0.6)	-4 (1.2)
Montreal	35 (2.1)	23 (1.4)	13 (2.9)	18 (2.9)	9 (1.0)	**10** (1.6)	16 (0.7)	-2 (1.3)
Ottawa	37 (2.9)	27 (2.5)	9 (5.8)	14 (5.3)	7 (1.6)	6 (1.3)	16 (0.6)	-2 (1.1)
Durham	41 (3.0)	29 (2.0)	9 (4.9)	12 (4.2)	8 (1.9)	6 (1.1)	16 (0.7)	0 (1.2)
York	45 (2.7)	**32** (1.2)	7 (1.5)	11 (1.8)	8 (1.7)	5 (0.9)	17 (0.7)	0 (1.1)
Toronto	41 (2.4)	25 (1.2)	**18** (3.4)	21 (2.8)	9 (1.6)	6 (0.9)	17 (0.7)	2 (1.0)
Peel	43 (2.4)	28 (1.3)	12 (3.3)	17 (3.4)	9 (1.7)	6 (0.9)	17 (0.9)	1 (1.2)
Halton	44 (2.9)	28 (1.4)	12 (2.7)	15 (2.3)	9 (2.2)	6 (1.0)	17 (0.7)	2 (1.0)
Hamilton	43 (2.4)	27 (2.3)	14 (3.8)	16 (3.4)	10 (1.9)	7 (1.0)	16 (0.7)	1 (1.1)
Niagara	44 (2.7)	28 (2.7)	10 (3.2)	13 (3.2)	9 (1.5)	6 (0.7)	17 (0.6)	2 (1.0)
Waterloo	45 (2.8)	30 (1.7)	8 (2.3)	13 (2.9)	9 (1.5)	6 (0.8)	16 (0.6)	0 (1.2)
Windsor	**47** (2.9)	25 (3.4)	14 (3.2)	19 (2.8)	11 (1.7)	7 (0.7)	**18** (0.7)	2 (1.0)
Sarnia	45 (3.1)	29 (2.3)	10 (3.3)	13 (3.1)	**13** (1.4)	**10** (0.9)	17 (0.6)	1 (1.1)
London	44 (2.9)	27 (2.0)	10 (3.0)	14 (3.8)	10 (2.5)	7 (1.9)	16 (0.7)	1 (1.1)
Sudbury	39 (2.8)	**32** (1.6)	6 (0.7)	10 (1.2)	NA[h]	NA	14 (0.8)	-5 (1.1)
Sault Sainte Marie	39 (2.3)	**32** (1.1)	5 (2.0)	8 (2.6)	NA	NA	14 (0.8)	-3 (1.2)
Winnipeg	34 (3.6)	25 (2.3)	6 (1.5)	11 (1.7)	6 (0.9)	5 (0.8)	15 (1.1)	-7 (1.4)
Regina	30 (5.5)	21 (4.8)	9 (1.6)	14 (2.2)	7 (1.8)	5 (1.3)	13 (0.9)	**-8** (1.2)
Saskatoon	33 (2.5)	24 (1.9)	8 (1.5)	14 (1.2)	NA	NA	13 (0.9)	**-8** (1.1)
Calgary	36 (2.0)	24 (1.8)	14 (3.4)	**25** (3.0)	8 (2.4)	7 (2.1)	12 (0.8)	-3 (0.9)
Edmonton	37 (1.9)	23 (1.5)	12 (2.9)	23 (3.4)	7 (2.1)	8 (3.1)	12 (0.7)	-6 (1.1)
Vancouver	29 (1.2)	21 (2.0)	12 (1.7)	16 (1.8)	5 (0.5)	5 (0.8)	14 (0.7)	6 (0.8)
Combined[g]	38 (6.0)	27 (3.3)	10 (3.1)	15 (4.3)	8 (1.9)	6 (1.7)	15 (1.8)	-1 (3.4)

[a] Ozone concentrations were calculated using the imputed daily rolling average of the maximum eight hours.
[b] SD: standard deviation of 12-year annual averages.
[c] NO2 concentrations were calculated using the imputed 24-hour daily average.
[d] PM2.5 concentrations were calculated using the imputed unadjusted 24-hour daily average.
[e] Temperature concentrations were calculated using 24-hour daily average.
[f] Warm (April to September); Cold (October to March).
[g] Average (SD) over 24 cities.
[h] PM2.5 concentrations are not available due to limited data in Sudbury, Sault Sainte Marie, and Saskatoon.

Table S2. City-specific annual average Pearson’s correlations between Ozone, NO2, and PM2.5 for 2001-2012

City[a]	Ozone & NO2 (SD)			Ozone & PM2.5 (SD)			PM2.5 & NO2 (SD)		
	Warm[b]	Cold[b]	Year[b]	Warm[b]	Cold[b]	Year[b]	Warm[b]	Cold[b]	Year[b]
Halifax	-0.1 (0.13)	-0.2 (0.15)	-0.1 (0.09)	0.3 (0.17)	0.1 (0.21)	0.2 (0.18)	0.1 (0.19)	0.1 (0.14)	0.1 (0.16)
Saint John	0.3 (0.15)	0.0 (0.21)	0.1 (0.14)	0.3 (0.09)	0.1 (0.16)	0.2 (0.08)	0.4 (0.15)	0.5 (0.16)	0.4 (0.13)
Quebec City	0.2 (0.08)	-0.3 (0.12)	-0.2 (0.06)	0.5 (0.13)	-0.3 (0.11)	0.1 (0.13)	0.4 (0.09)	**0.8 (0.08)**[d]	0.6 (0.09)
Montreal	0.3 (0.10)	-0.2 (0.11)	-0.2 (0.07)	0.6 (0.08)	-0.3 (0.10)	0.2 (0.15)	0.4 (0.08)	**0.8 (0.04)**	0.6 (0.10)
Ottawa	0.3 (0.10)	-0.3 (0.10)	-0.2 (0.07)	0.6 (0.11)	-0.2 (0.09)	0.3 (0.10)	0.3 (0.13)	**0.7 (0.09)**	0.4 (0.09)
Durham	0.3 (0.15)	-0.3 (0.18)	-0.1 (0.09)	**0.7 (0.14)**	-0.1 (0.22)	0.5 (0.10)	0.4 (0.16)	0.6 (0.23)	0.4 (0.17)
York	0.3 (0.09)	-0.3 (0.15)	-0.2 (0.08)	**0.7 (0.07)**	0.0 (0.15)	0.5 (0.10)	0.3 (0.07)	0.6 (0.09)	0.3 (0.06)
Toronto	0.3 (0.08)	-0.2 (0.13)	-0.1 (0.06)	**0.7 (0.07)**	-0.1 (0.14)	0.5 (0.08)	0.4 (0.08)	0.6 (0.06)	0.4 (0.06)
Peel	0.1 (0.08)	-0.3 (0.11)	-0.2 (0.05)	**0.7 (0.07)**	-0.1 (0.12)	0.5 (0.09)	0.3 (0.07)	0.6 (0.07)	0.3 (0.06)
Halton	0.2 (0.07)	-0.3 (0.13)	-0.1 (0.07)	**0.7 (0.07)**	-0.1 (0.14)	0.5 (0.10)	0.3 (0.11)	0.6 (0.09)	0.4 (0.09)
Hamilton	-0.1 (0.10)	-0.3 (0.12)	-0.3 (0.08)	0.6 (0.11)	-0.1 (0.14)	0.5 (0.11)	0.3 (0.13)	0.6 (0.12)	0.3 (0.12)
Niagara	0.0 (0.17)	-0.1 (0.16)	-0.2 (0.10)	**0.7 (0.08)**	0.0 (0.12)	0.5 (0.08)	0.1 (0.17)	0.4 (0.23)	0.1 (0.18)
Waterloo	0.2 (0.15)	-0.2 (0.14)	-0.2 (0.08)	**0.7 (0.10)**	0.0 (0.14)	0.5 (0.10)	0.2 (0.15)	0.5 (0.13)	0.2 (0.11)
Windsor	0.2 (0.12)	-0.2 (0.15)	-0.3 (0.06)	**0.7 (0.07)**	0.0 (0.15)	0.5 (0.10)	0.3 (0.09)	0.5 (0.09)	0.2 (0.08)
Sarnia	0.5 (0.06)	-0.3 (0.15)	0.0 (0.10)	**0.7 (0.08)**	-0.2 (0.15)	0.4 (0.10)	0.6 (0.08)	**0.7 (0.08)**	0.5 (0.09)
London	0.4 (0.09)	-0.1 (0.16)	-0.2 (0.06)	0.6 (0.18)	0.1 (0.14)	0.5 (0.07)	0.3 (0.18)	0.5 (0.26)	0.2 (0.18)
Sudbury	0.2 (0.12)	0.1 (0.04)	-0.1 (0.05)	NA[e]	NA	NA	NA	NA	NA
Sault Sainte Marie	0.2 (0.12)	-0.1 (0.22)	-0.1 (0.10)	NA	NA	NA	NA	NA	NA
Winnipeg	0.3 (0.12)	-0.2 (0.17)	-0.2 (0.09)	0.3 (0.13)	-0.2 (0.12)	0.3 (0.14)	0.3 (0.13)	0.4 (0.17)	0.2 (0.15)
Regina	0.1 (0.16)	-0.1 (0.23)	-0.2 (0.15)	0.2 (0.21)	-0.1 (0.16)	0.2 (0.15)	0.0 (0.17)	0.2 (0.17)	0.0 (0.17)
Saskatoon	0.1 (0.18)	-0.1 (0.19)	-0.3 (0.13)	NA	NA	NA	NA	NA	NA
Calgary	0.1 (0.13)	-0.4 (0.10)	-0.5 (0.07)	0.3 (0.13)	-0.5 (0.10)	0.0 (0.18)	0.3 (0.17)	0.5 (0.11)	0.2 (0.15)
Edmonton	0.2 (0.12)	-0.4 (0.11)	-0.5 (0.07)	0.3 (0.14)	-0.4 (0.13)	-0.1 (0.13)	0.4 (0.11)	0.5 (0.10)	0.4 (0.11)
Vancouver	0.1 (0.14)	-0.5 (0.13)	-0.4 (0.11)	0.2 (0.11)	-0.6 (0.09)	-0.1 (0.17)	**0.7 (0.08)**	0.6 (0.08)	0.5 (0.10)
Combined[c]	0.2 (0.13)	-0.2 (0.14)	-0.2 (0.13)	0.5 (0.18)	-0.1 (0.18)	0.3 (0.20)	0.3 (0.14)	0.5 (0.16)	0.3 (0.16)

[a] Cities are ordered geographically from east to west.

[b] Warm (April to September); Cold (October to March); Year (January to December).

[c] Average over 24 cities.

[d] Bold indicates high correlation ≥ 0.7.

[e] Pearson’s correlation is not available due to limited PM2.5 data in Sudbury, Sault Sainte Marie, and Saskatoon.

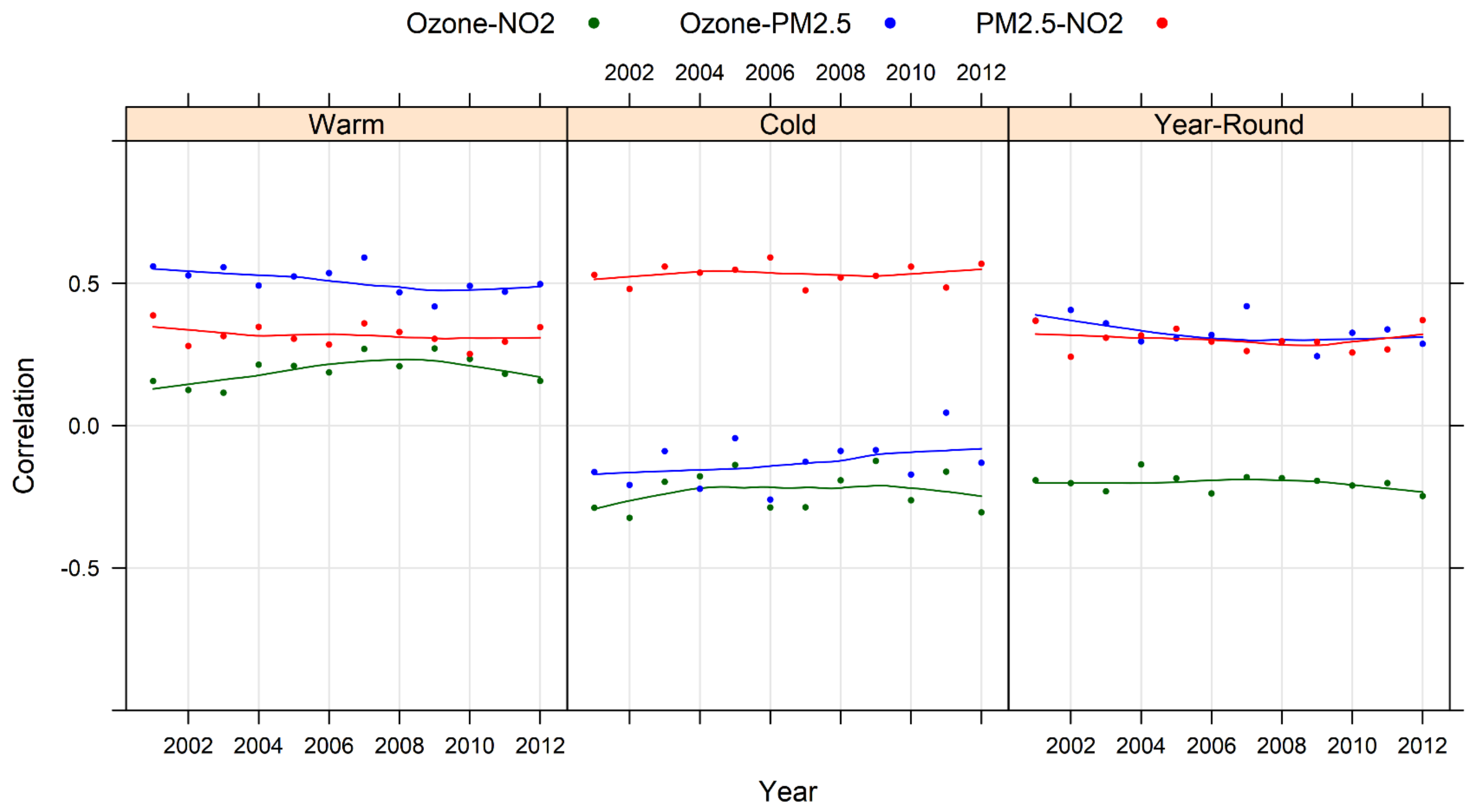

Figure S2: Annual Pearson correlation between three air pollutants for 2001-2012 by season: (left) warm season (April to September); (middle) cold season (October to March); and (right) year-round (January to December). In color, (green) correlation between ozone and NO2, (blue) correlation between ozone and PM2.5, and (red) correlation between NO2 and PM2.5. Lines are generated by a LOESS smoother (span = 0.75, degree= 1)

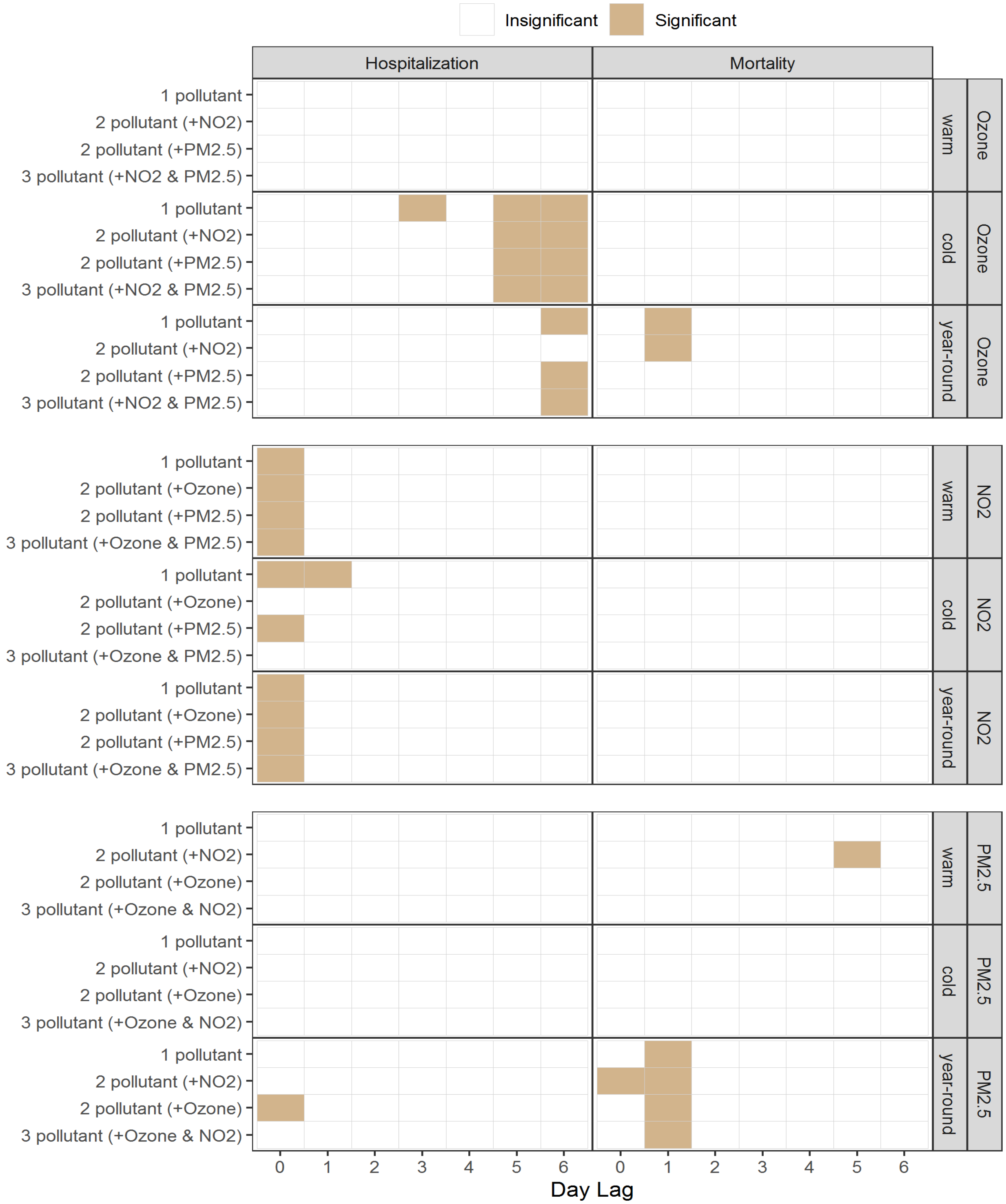

Figure S3: Diagram on significance of associations between 3 air pollutants and circulatory hospitalization and mortality, respectively, by model, season and lag: (a) 4 models of 1-, 2-, or 3-pollutants together; (b) 3 seasons of warm (Apr to Sept), cold (Oct to Mar) and year-round (Jan to Dec); and (c) 7 lags of 0- to 6-day lagged air pollutant. The highlighted indicate significance, and the blank does insignificance
